# Factors affecting detection and quantification of *Schistosoma haematobium* eggs in pooled urine samples

**DOI:** 10.1371/journal.pntd.0014407

**Published:** 2026-06-01

**Authors:** Abraham Degarege, Bruno Levecke, Christopher R. Bilder, David M. Brett-Major, Abebe Animut, Yohannes Negash, M. Jana Broadhurst, Tzeyu L. Michaud, Berhanu Erko

**Affiliations:** 1 Department of Epidemiology, College of Public Health, University of Nebraska Medical Center, Omaha, Nebraska, United States of America; 2 Department of Translational Physiology, Infectiology and Public Health, Ghent University, Ghent, Belgium; 3 Department of Statistics, University of Nebraska-Lincoln, Lincoln, Nebraska, United States of America; 4 Aklilu Lemma Institute of Health Research, Addis Ababa University, Addis Ababa, Ethiopia; 5 Department of Pathology, Microbiology and Immunology, University of Nebraska Medical Center, Omaha, Nebraska, United States of America; 6 Department of Health Promotion, College of Public Health University of Nebraska Medical Center, Omaha, Nebraska, United States of America; Erasmus MC, University Medical Center Rotterdam, NETHERLANDS, KINGDOM OF THE

## Abstract

**Background:**

Building on our previous work showing that pooled urine testing can rapidly detect *Schistosoma haematobium* infections but has limited sensitivity in low-intensity settings or with large pool sizes, this study evaluated how urine volume, pool size, infection intensity, and diagnostic method affect the detection and quantification of *S. haematobium* eggs in pooled samples.

**Methods:**

Between July 2022 and April 2023, 2,134 urine samples from school-age children living in three regional states of Ethiopia were individually examined by deploying urine filtration microscopy (UFM). Subsequently, 5, 10, 20 and 40 individual samples were strategically pooled and examined by deploying UFM and Fluke Catcher (FC) and varying volumes of urine (10, 20 and 30 mL).

**Results:**

UFM was significantly more sensitive than FC for detecting *S. haematobium* eggs in pooled urine samples ($\hat{\beta }$ =0.83, *p* < 0.001). There were significant interactions between the mean log of urine egg count (UEC) and volume of urine ($\hat{\beta }$ =0.01, *p* = 0.021) or pool size ($\hat{\beta }$ =-0.02, *p* = 0.007) for detecting *S. haematobium* eggs in pooled samples. The odds of detecting eggs in pooled samples in which the mean UEC of individual samples equaled 10 eggs/mL were 0.78 (95% CI:0.67-0.90) and 0.60 (95% CI:0.51-0.76) times lower when 10 mL rather than 20 mL and 30 mL was examined, respectively. There was a moderate or strong positive correlation between the mean egg count of pooled samples and the average egg count of the individual samples making the pools when pool sizes were 5, 10 or 20 (r ≥ 0.5).

**Conclusions:**

The diagnostic sensitivity of pooled testing for diagnosing *S. haematobium* infection is affected by a complex interplay between the pool size, the volume of urine examined, the mean UEC and the diagnostic method. The sensitivity of the pooled testing strategy may increase with larger urine volumes, higher infection intensity, and when UFM (rather than FC) is used for testing. However, sensitivity could decrease as the pool size increases. Use of UFM, larger urine volumes and smaller to moderate pool sizes could improve detection of *S. haematobium* infection in surveillance and mass deworming programs in low-intensity settings.

## Author summary

*Schistosoma haematobium* is a parasite that causes a serious disease affecting millions of people in sub-Saharan Africa and the Middle East. The infection is usually detected by looking for parasite eggs in urine samples under a microscope. However, testing each person individually is time-consuming and costly, especially during large community surveys or mass treatment campaigns. This study explored a faster and more affordable approach called pooled testing, where urine samples from several people are combined and tested together. We collected urine samples from school-aged children in parts of Ethiopia where this infection is common. Each sample was tested individually and in pools of 5, 10, 20, or 40 people, using urine filtration microscopy (UFM) and the Fluke Catcher (FC) methods while varying the urine volume tested (10, 20, or 30 mL). We found that UFM was more accurate than the FC for detecting infection in pooled samples. The pooled testing method worked best when the infected person had more parasite eggs, particularly when larger urine volumes were examined, and smaller pool sizes were used. These results suggest that pooled urine testing could be a practical solution for rapidly monitoring *S. haematobium* infection in resource-limited communities. Optimizing pool size, urine volume, and testing methods can improve accuracy and support community screening and deworming programs in resource-limited areas.

## Background

Urogenital schistosomiasis is a parasitic disease caused by the flatworm *Schistosoma haematobium* and affects more than 112 million people worldwide, particularly in sub-Saharan Africa and the Middle East [1–3]. In children, the disease can cause hematuria (blood in urine), anemia, malnutrition, and cognitive deficiency, while in adults, complications include kidney damage, fibrosis in the bladder, narrowing of the ureters, genital lesions, and bladder cancer [1–3]. In high endemicity settings, children often suffer more severe disease [4].

To reduce transmission of schistosomiasis and the associated morbidities, school-age children living in endemic regions periodically receive a single oral dose of 40 mg/kg praziquantel through mass drug administration (MDA) programs [5,6]. Decisions on whether to initiate such large-scale deworming programs in a specific area and at which frequency (annually *vs.* bi-annually) are based on the disease prevalence [5,6]. Once initiated, there is also a need to periodically re-assess the prevalence to verify if scaling down the frequency or even stopping the administration of drugs is justified [5,6]. To this end, the availability of a reliable and sustainable *S. haematobium* diagnostic strategy remains a top priority in endemic areas. Screening of individual urine samples remains the current diagnostic strategy to detect cases and subsequently draw conclusions. Yet, this strategy is resource intensive [1–4].

A potential cost-saving strategy involves pooling individual samples and applying a cascaded pooled testing (CPT) strategy. In such a testing strategy, samples are first tested as a pool, and samples from positive pools are then tested individually to identify the positive cases [7,8]. Conversely, if a pool tests negative, all individuals within that pool are considered negative [7,8].

CPT has a very long history for a diverse set of applications. While Robert Dorfman [7] is usually given credit as the first to suggest the process, there are records of CPT being used as far back as 1915 [9]. Since then, CPT has been refined and widely used across applications including SARS-CoV-2 testing [10], blood donation screening [11], sexually transmitted infection detection [12], and animal disease testing [13]. General textbook summaries of CPT are available from a variety of sources [9,14,15] and from general overview papers [9,16,17].

CPT has been shown to reduce the number of tests, and hence the operational costs, of testing programs for infectious diseases, including helminthiasis [18–21]. However, the application of CPT to helminth egg detection in urine has been limited. Previously, we evaluated the performance of a CPT strategy (pools of 5, 10 and 20 individual samples) in determining the presence and the intensity of *S. haematobium* infection (by counting the number of eggs in 10 mL of urine) at the population level applying the urine filtration method (UFM) [22,23]. These studies showed that pooled testing strategy can provide a rapid assessment of infection intensity; however, its sensitivity for estimating population prevalence may be reduced in low intensity settings, particularly as pool size increases [22,23]. Thus, more studies are needed to determine when and how pooling can be optimally implemented to monitor *S. haematobium* infections in the context of MDA programs.

Potential measures to improve the performance of this strategy are (i) to screen an increased volume of urine and (ii) to apply more sensitive diagnostic tests. UFM is the standard method for the diagnosis of *S. haematobium* infection. By this method, urine samples are filtered through nylon or polycarbonate membranes followed by microscopic examination of the filter for parasite eggs [1–3]. UFM shows reduced sensitivity for detecting low intensity *S. haematobium* infection [1–3]. The Fluke Catcher (FC) is an alternative diagnostic method that allows for examining a larger volume of urine. This method has been deployed in veterinary medicine to detect fluke eggs (e.g., *Fasciola*) in the stool of livestock and consists of a stack of three filters with different mesh sizes [24–26]. Recently, the FC method has shown high sensitivity for detecting *Fasciola hepatica* eggs in human stool [27], but its performance for detecting and quantifying *S. haematobium* eggs in urine is not known.

The aim of our study was to explore how pooled urine testing can be optimally implemented for monitoring *S. haematobium* infections in the context of an MDA program. For this, we designed a set of laboratory experiments to examine the impact of (i) volume of urine (10, 20 and 30 mL), (ii) pool size (5, 10, 20, and 40 individual samples) (iii) intensity of infection, and (iv) diagnostic method (UFM *vs.* FC) on the ability of a pooled testing strategy to detect and quantify *S. haematobium* eggs in pooled urine samples.

## Methods

### Ethics statement

This study was approved by the institutional review boards of the University of Nebraska Medical Center, USA (Ref. No. IRB #0875-21-EP) and Aklilu Lemma Institute of Health Research, Ethiopia (Ref. No. ALIPB IRB/63/2014/2021). The district health office, school authorities, and teachers were informed about the purpose and procedures of the study. Written informed consent without a signature was obtained from parents or guardians of children who assent to participate in the study. As the study population was members of a distinct cultural group or community in which signing forms is not the norm, the institutional review board approved a waiver of signed consent for this study. Accordingly, parents or legal guardians received written information describing the study objectives, procedures, risks, and benefits. Verbal informed consent was obtained in lieu of written signatures, with a study team member serving as witness to confirm that the information was explained, questions were answered, and participation was voluntary. A praziquantel treatment (40 mg/kg body weight) was provided free of charge to all children infected with *S. haematobium* (based on UFM using 10 mL of urine). Praziquantel was administered by participating health workers from the local area.

### Study design

We conducted an experimental study using urine specimens collected from Ethiopian children between June 2022 and April 2023. Urine samples were collected at baseline and one month after praziquantel was administered to children infected with the parasite based on urine testing results. All urine samples were individually examined for *S. haematobium* eggs by UFM. Urine samples with *S. haematobium* eggs detected were pooled with negative samples and examined using UFM and FC at the Medical Parasitology Laboratory of Aklilu Lemma Institute of Health Research of Addis Ababa University.

### Study population and study area

The study was conducted among school-age children (5–15 years of age) living in the three Ethiopian Regional States (Afar, Benishangul-Gumuz and Gambella) where urogenital schistosomiasis is endemic (prevalence of the disease in Afar: 2.3% to 52.0%; in Benishangul-Gumuz: 35.9% to 57.8%; in Gambella: 35.9% to 43.8%) [22,23,28–30]. The Global Positioning System coordinates of the study areas were as follows: Afar 11.8167, 41.4167; Benishangul Gumuz 10.6390, 35.7330; Gambella 7.6184, 34.6893. A total of 15 villages were selected for this study, including seven villages from the Afar region, seven from the Benishangul-Gumuz region, and one from the Gambella region. These locations were chosen based on reports of *S. haematobium* transmission or expectations of its presence as recommended by local community leaders. Children living in these study regions are sporadically treated with praziquantel as part of the MDA program. However, the children who participated in this study did not receive PZQ within three months of the data collection period.

### Sample size

The sample size for each study region was estimated using published sample size tables from a review article that summarize the minimum number of samples required for sensitivity and specificity analyses [31]. These estimates were calculated using Power Analysis and Sample Size (PASS) software, incorporating specified confidence interval levels, statistical power, and disease prevalence assumptions [32]. We previously found a 51.7% sensitivity of the hierarchical pooling technique for detecting *S. haematobium* infection using UFM with 10 mL urine (H_o_) [23]. In order to test the hypothesis that the sensitivity of the pooled testing is at least 60% (H_a_) when the volume of urine examined is > 10 mL with a power of 80% (type II error of 20%) and type I error level of 5%, we needed to screen a minimum of 995 children in Afar and 995 children in Benishangul-Gumuz where the prevalence of *S. haematobium* infection was estimated to be 20%, and 498 in Gambella where the prevalence of *S. haematobium* infection was estimated to be 40% [22,23,28–30]. Due to the low prevalence rate in some villages in the Afar region, we proposed collecting samples from an additional 100 (10% of 995) children in Afar to ensure the collection of an adequate number of positive samples to assess sensitivity. The total sample size estimated for this study was 2,588.

### Urine sample collection and processing

We first briefed the school directors and village officials on the study’s objectives. Subsequently, the administrators of the villages informed the villagers about the aim of the research. Then, the study team recruited children who agreed to participate, and whose parents or guardians approved their participation (see ethics statement). Eligible children were recruited using consecutive (convenience) sampling from predefined community collection points, including nearby health posts, schools, and open field stations within the villages. Consent discussion was conducted privately in nearby health posts, schools, or designated open fields within the villages, where the study objectives, procedures, potential benefits, and risks were explained. Following consent and assent, participating children were asked to provide at least 80 mL in labeled 200 mL plastic containers between 10:00 am and 3:00 pm. Urine samples were collected before recording demographic data such as age and gender to minimize bias. From each 80 mL urine sample, 10 mL was filtered using a polycarbonate filter membrane (13 mm diameter and 12–20 μm pore size) and examined for *S. haematobium* eggs at the collection sites or the nearby health posts using UFM. Prior to subsampling for filtration and egg quantification, the individual urine samples were thoroughly homogenized by gentle inversion and agitation to minimize sedimentation and ensure an even distribution of eggs within each aliquot. UFM results were shared confidentially with participating children and their parents or guardians at the sample collection sites, with support from local health personnel or nurses and school staff or village officials when needed. Children whose urine tested positive for *S. haematobium* eggs were treated with Praziquantel by participating health workers. The remaining 70 mL of urine was transferred to a vial containing 0.7 mL formalin (37% formaldehyde) and transported to the Medical Parasitology Laboratory of Aklilu Lemma Institute of Health Research of Addis Ababa University.

**Fig 1:**
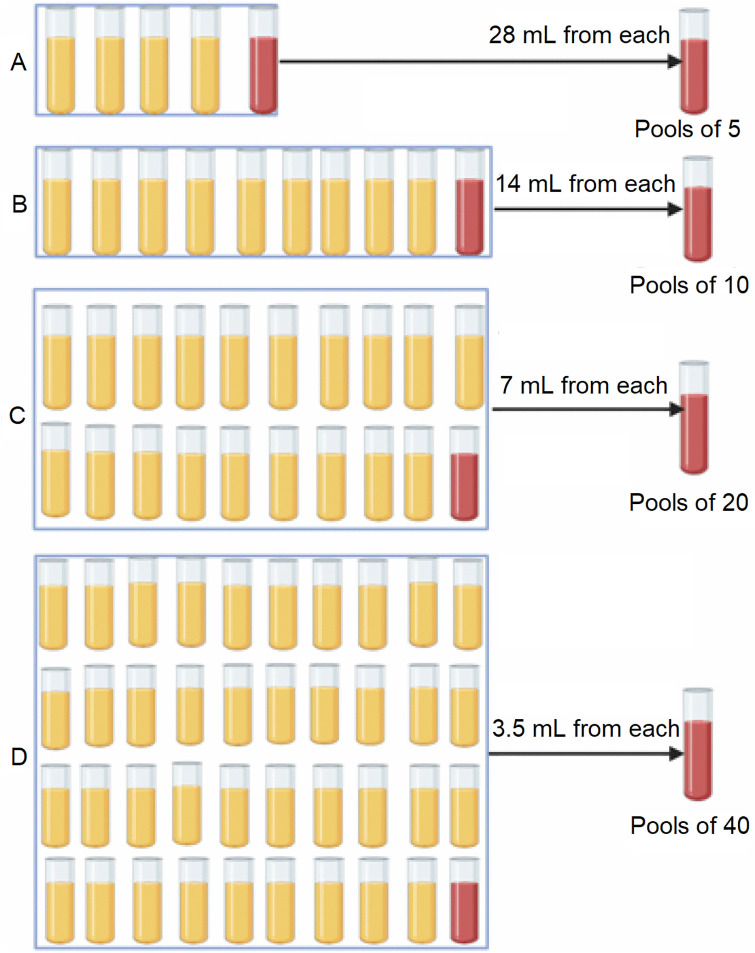
The applied strategy to experimentally make pools of 5, 10, 20 and 40 individual samples. Formalin-fixed individual urine samples were arranged in groups of 5 (Panel A), 10 (Panel B), 20 (Panel C) and 40 (Panel D) individual urine samples containing one positive sample, while the remaining samples were negative. Then, an aliquot, proportional to the number of individual samples, was transferred into a new vial, resulting in pooled samples with a fixed total volume of 140 mL. Created in BioRender. Mengist, A. (2026) https://BioRender.com/ad0khd9.

Experimental pooling of urine samples was carried out in the laboratory, approximately 1–3 weeks after their collection in the field. We first re-tested 10 mL of the formalin-fixed urine samples from each child using UFM after the field data collection. An experienced microscopist with over 15 years of expertise in using UFM for *S. haematobium* diagnosis performed the examination. Based on the UFM results obtained at the collection site and in the laboratory, each individual urine sample was declared either positive (any egg was found at least once) or negative for *S. haematobium* eggs (no eggs were found on both occasions). Then, plastic vials containing formalin-fixed individual urine samples were arranged in groups of 5, 10, 20, and 40 samples, respectively (**Fig 1**). In each group, one positive sample was included, while the remaining samples were negative. The positive sample included in each pool was selected at random from the set of known positive specimens and was not chosen based on infection intensity. Then, an aliquot of each sample within a group was transferred into a new vial, resulting in pooled samples with a fixed total volume of 140 mL (pools of 5: 28 mL per sample; pools of 10: 14 mL per sample; pools of 20: 7 mL per sample and pools of 40: 3.5 mL per sample). Finally, after shaking/mixing the pooled samples to ensure uniform distribution of the eggs of the parasite, an aliquot of 10 mL, 20 mL, and 30 mL of each pooled urine sample were tested deploying UFM [33,34] and FC [24]. The pooled samples were mixed by gentle inversion and agitation immediately before aliquots were taken for filtration and egg quantification. One month after treatment, children who tested positive for *S. haematobium* infection using UFM in the field and were treated with praziquantel (40 mg/kg body weight) were re-examined using the same methodology as described above. During the second-round survey, children who tested negative for *S. haematobium* using UFM in the field but were subsequently found positive in laboratory testing received treatment.

### Data management and statistical data analysis

The raw data were first entered into an Excel spreadsheet and carefully checked for errors, incorrect values, or missing data. Since missing values accounted for less than 1% and were related to age, sex, or individual sample test results, incomplete entries were removed. Inaccurate or inconsistent values were corrected by cross-referencing with the source documents. After addressing any inaccuracies, inconsistencies, or missing data, the cleaned dataset was transferred to R for analysis [35].

The data analysis consisted of three consecutive steps. First, we provided the overall prevalence and intensity of *S. haematobium* infections and their variation across age, sex, and geographical area. Second, we examined the impact of (i) the volume of urine, (ii) the pool size, (iii) the intensity of infection, and (iv) the diagnostic method on the diagnostic sensitivity (the ability to detect a single infected individual in pooled samples) and egg count. Finally, we assessed and compared the infection intensity at a population level between an individual and a pooled testing strategy.

The overall prevalence of *S. haematobium* is the proportion of the screened children that tested positive at least once either in the field or at the laboratory. To assess factors associated with the prevalence of *S. haematobium* infection, we fitted logistic regression model. To assess factors associated with the intensity of *S. haematobium* infection, we fitted zero-inflated negative binomial (ZINB) regression models to account for overdispersion and excess zero egg counts. Age, gender, and village of residence were included as covariates. Stata was used to find the zero-inflated negative binomial model [36].

The overall diagnostic sensitivity was determined by calculating the proportion of the pooled urine samples that tested positive for *S. haematobium* eggs. A line graph was used to examine the changes in the sensitivity of the pooled test across the volume of urine examined; pool size; diagnostic methods; and intensity levels of infection (level 1: mean urine egg count (UEC) ≤ 25th percentile, level 2: 25^th^ percentile < mean UEC ≤ 50^th^, level 3: 50^th^ percentile < mean UEC ≤ 75^th^ percentile, levels 4: > 75th percentile). A logistic regression model was fit to the data to estimate the sensitivity. Included in this model were: urine volume (10, 20 and 30 mL), pool size (5, 10, 20 and 40), UEC, and diagnostic method (UFM and FC) as main effects, a urine volume and UEC interaction, a pool size and UEC interaction, and random effect for pool ID. Pool ID was included as a random effect because urine samples from the same individual were examined using 10 mL, 20 mL, and 30 mL urine volumes, as well as in pools of 5, 10, 20, and 40 samples. This model was chosen through a model building process. To interpret the interactions, we performed pairwise comparisons across different combinations. To correct for multiple comparisons, we deployed the Tukey method. Based on this model, we determined the combinations of the volume of urine, diagnostic method and pool size that would allow for an estimated diagnostic sensitivity of at least 95% across different scenarios of infection intensity.

Egg counts from pooled urine samples were modeled using ZINB regression to account for overdispersion and excess zeros. Pool size (5,10,20,40), the mean egg count of constituent individual samples, diagnostic method (UFM vs FC) and their interaction were included as covariates to estimate factors associated with egg count/intensity in pooled urine samples. Stratified analyses by urine volume (10, 20, and 30 mL) were conducted to evaluate differences in dispersion across strata. Estimates/results from ZINB are reported as incidence rate ratios (IRRs), with 95% confidence intervals.

A paired t-test was used to assess the differences in the average UEC between the pooled samples and members of the pooled samples (i.e., the individual samples making the pools). We also explored the relationship between the UEC in the pool and the average UEC across the corresponding individual samples using a scatter plot and a linear regression model to fit the data to understand the relationship. Pearson correlation was used to examine the strength of agreement in UEC in the pool and mean UEC across the corresponding individual samples [37]. The data were analyzed using the R statistical software package [38].

## Results

### Prevalence and intensity of *S. haematobium* infections

**Fig 2:**
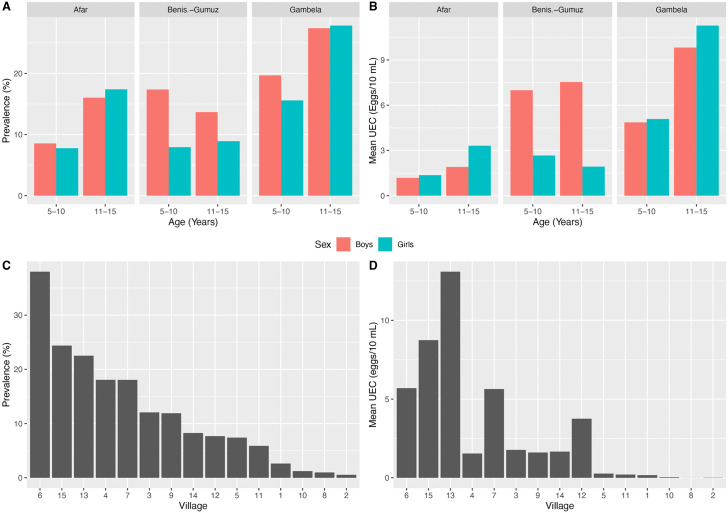
The prevalence and arithmetic mean UEC of *S. haematobium* infection among 2,634 Ethiopian school-aged children by regions, sex, age groups and villages (1 Admegala; 2. Ambash 3. Andade; 4: Buri; 5: Erimberte; 6: Gabole; 7: Haldebe; 8: Hassoba; 9: Kusra; 10 Akandayu; 11: DuleHode; 12: Kumruk; 13: Salima; 14 Shetalo; 15: Village.

A total of 2,634 children (ages ranging from 5 to 15 years, 41.7% girls) were sampled at baseline across three regions and 15 villages (Afar: nine villages; Benishangul-Gumuz: five villages and Gambella: one village). Overall, 14.05% out of the 2,634 children tested positive for *S. haematobium* eggs on at least one occasion (i.e., when tested in the field or at the laboratory) when deploying UFM. Most of the infection intensities were light (1–49 eggs per 10 ml of urine) in nature (83.50%). Overall, infections were more prevalent in older children (5–10 years: 12.15% *vs.* 11–15 years: 16.19%), boys (boys: 15.04% *vs.* girls: 12.66%) and in Gambella (Gambella: 24.37% *vs.* Benishangul-Gumuz: 12.36% *vs.* Afar: 11.64%). Infections were observed in all villages, where the prevalence ranged from 0.55% (Ambash in Afar) to 38.00% (Gabole in Afar). **Fig 2** summarizes the prevalence and the mean UEC of *S. haematobium* infection across age, sex, regions, and villages. S1 Fig shows distribution of *S. haematobium* egg counts (log eggs/10 mL urine) among study participants, stratified by study region.

In the logistic regression model, an increase in age was associated with an increase in the odds of *S. haematobium* infection (OR = 1.06, 95% CI: 1.02–1.10, p = 0.002), whereas village and gender were not associated with the odds of *S. haematobium* infection (S1 Table 1). In zero-inflated negative binomial models, there was moderate evidence for village being associated with *S. haematobium* infection intensity (IRR = 1.09, 95% CI: 1.01–1.17, p = 0.025), whereas there was not sufficient evidence that age and gender were associated with infection intensity (S1 Table).

### Factors affecting the diagnostic sensitivity

**Fig 3:**
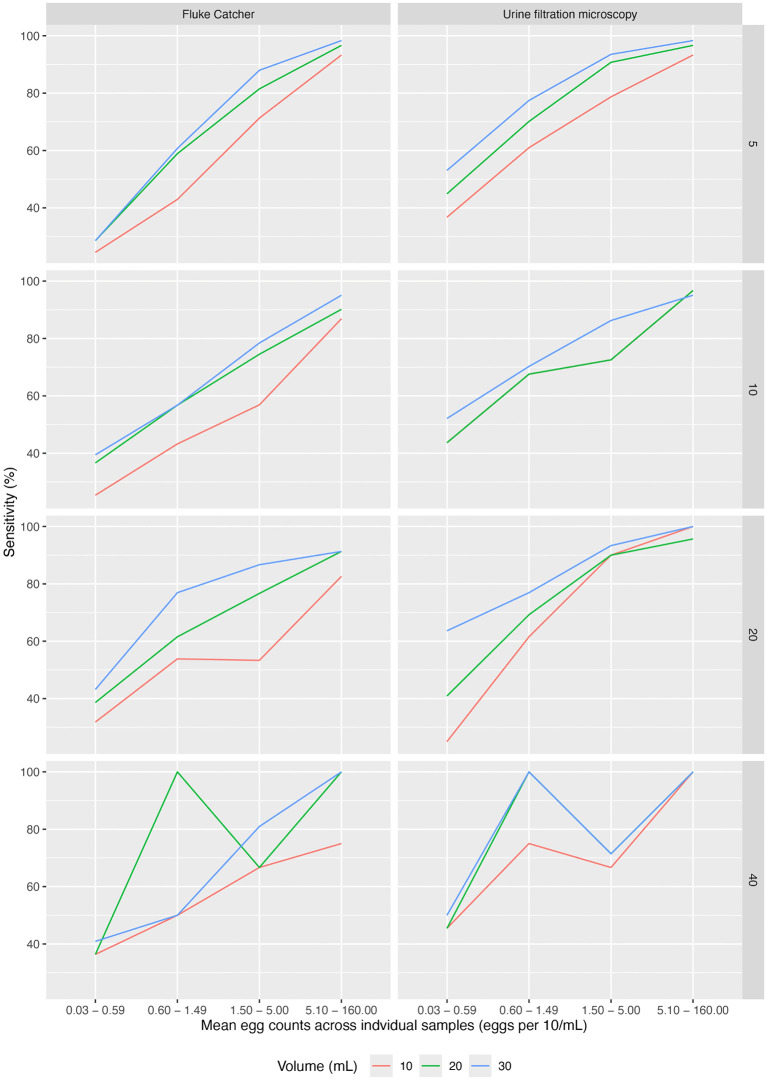
The observed sensitivity by diagnostic methods (urine filtration microscopy and Fluke Catcher), volume of urine examined (10, 20 and 30 mL), pool size (5, 10, 20 and 40) and mean UEC across the individual samples making the pools. The red line, representing sensitivity based on the examination of 10 mL using UFM for a pool size of 10, overlaps with other lines.

Overall, the diagnostic sensitivity tends to increase as a function of the intensity level of infection and the volume of urine examined. The sensitivity tends to be higher when using the UFM method compared to the FC method. Fig 3 summarizes the diagnostic sensitivity across the volume of urine, the pool size, the diagnostic method, and the intensity of infection.

These observations were confirmed by the logistic regression model (**Table 1**). UFM was significantly more sensitive than FC for detecting *S. haematobium* eggs in pooled urine samples ($\hat{\beta }$ =0.83, p < 0.001). There were significant interactions between the mean log of urine egg count (UEC) and volume of urine ($\hat{\beta }$ =0.01, *p* = 0.021) or pool size ($\hat{\beta }$ =-0.01, *p* = 0.007) for detecting *S. haematobium* eggs in pooled samples. The odds of detecting eggs in pooled samples when 10 mL was examined and when the mean UEC of individual samples equaled 10 eggs/mL were 0.78 (95% CI: 0.67-0.90) and 0.60 (95% CI:0.51-0.76) times lower than when 20 mL and 30 mL are examined, respectively.

**Table 1:** Factors affecting the predicted diagnostic performance of pooled testing based on the logistic regression model.

	Estimate	Standard error	*p*-value
Intercept	0.17	0.16	0.277
Mean log of UEC (eggs per 10 mL of urine)	1.24	0.13	<0.001
Pool size	0.01	0.01	0.503
Volume of urine (mL)	0.06	0.01	<0.001
UFM *vs.* Fluke Cather	0.83	0.09	<0.001
Mean log of UEC x volume of urine	0.01	0.005	0.021
Mean log of UEC x pool size	-0.01	0.01	0.007

A subgroup analysis showed that the effects of both urine volume and pool size on the sensitivity of UFM for detecting *S. haematobium* in pooled samples varied by infection intensity. The effect of urine volume was greater in heavy-intensity infections (≥50 eggs/10 mL; β = 0.06, SE = 0.018, p < 0.001) than in light-intensity infections (<50 eggs/10 mL; β = 0.03, SE = 0.006, p < 0.001). Similarly, the negative effect of increasing pool size on sensitivity was more pronounced in heavy-intensity infections (β = −0.06, SE = 0.010, p < 0.001) compared with light-intensity infections (β = −0.03, SE = 0.006, p < 0.001).

**Fig 4:**
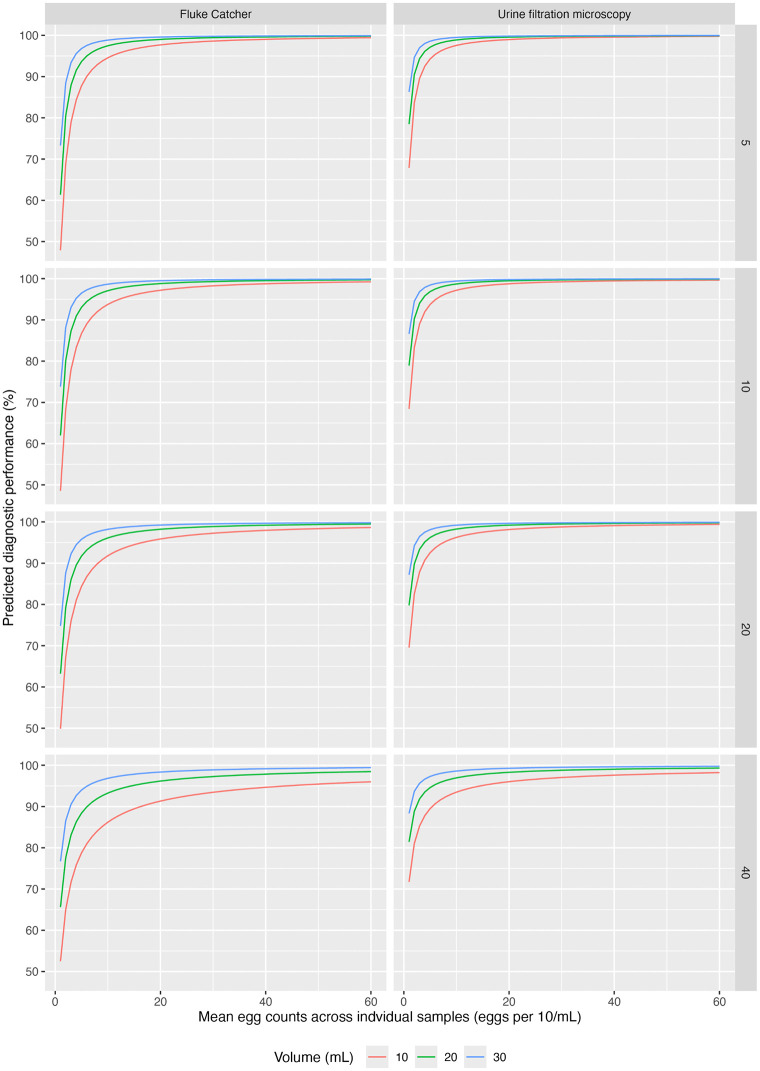
The predicted sensitivity across different combinations of volume, pool size, infection intensity and diagnostic method. These predicted values for diagnostic sensitivity were based on the logistic regression model in Table 2.

Based on the model predictions, we further explored which combination of urine volume, pool size and diagnostic method would allow for a diagnostic sensitivity of at least 95% across different scenarios of infection intensity (**Fig 4**).

**Table 2:** The lowest possible value of mean urine egg counts across the individual samples that can be detected with a probability of at least 95% for each combination of volume, pool size and diagnostic method.

Volume	Pool size	Urine Filtration	Fluke Catcher
10	5	6	11
	10	7	13
	20	8	17
	40	15	44
20	5	4	7
	10	4	7
	20	4	8
	40	6	15
30	5	3	4
	10	3	4
	20	3	5
	40	3	7

Overall, the lowest possible value of mean UEC across the individual samples that can be detected with an estimated probability of at least 95% for each combination of volume and pool size was lower when UFM was employed for testing than the FC. **Table 2** summarizes the lowest possible value of mean UEC across the individual samples (expressed as eggs in 10 mL urine) that can be detected with an estimated probability of at least 95% for each combination of volume, pool size and diagnostic method. These values are based on the logistic regression model described in **Table 1**.

Zero-inflated negative binomial regression model was fitted to account for overdispersion and excess zeros in pooled egg counts. In the negative binomial egg count, across all urine volumes (30, 20, and 10 mL), egg count or intensity of infection in pooled urine samples decreased consistently with an increase in pool size (IRR ≈ 0.98, p < 0.001) but increased with an increase in mean individual egg counts making the pools (IRR range: 1.05–1.06, p < 0.001) and when UFM used for the diagnosis than FC (IRR range: 1.29–1.49, p < 0.001) (S2 Table).

### Comparison of infection intensity at population level between the individual and pooled testing strategy

As would be expected, the mean UEC estimates of pooled samples tended to decrease with an increase in pool size because a smaller volume of the positive urine sample is examined. This is true for both UFM and FC. The average UEC per 10 mL in pooled samples tends to be lower than the average UEC per 10 mL of the individual samples making the pools regardless of the

pool size and diagnostic method. **Table 3** summarizes the mean UEC at the population level based on an individual and pooled testing strategy across the diagnostic method, different pool sizes and urine volume.

**Table 3:** Comparison of the urine egg count of individual and pooled samples.

Diagnostic test	Pool size (number of pools)	Volume of urine (mL) examined in pooled samples	Mean UEC per 10 mL		*p*-value for the difference from paired t-test
			Pools	Individuals	
UFM	5 (440)	10	7.38	7.68	0.652
		20	6.02	7.68	0.017
		30	5.24	7.68	<0.001
	10 (220)	10	4.49	5.19	0.182
		20	3.68	5.19	0.003
		30	3.29	5.19	<0.001
	20 (110)	10	2.06	3.48	<0.001
		20	2.27	3.48	0.009
		30	2.36	3.48	0.007
	40 (55)	10	1.89	1.98	0.852
		20	1.44	1.98	0.152
		30	1.61	1.98	0.261
FC	5 (439)	10	3.99	7.68	<0.001
		20	3.49	7.68	<0.001
		30	3.26	7.68	<0.001
	10 (220)	10	2.33	5.19	<0.001
		20	1.94	5.19	<0.001
		30	1.92	5.19	<0.001
	20 (110)	10	1.30	3.48	<0.001
		20	1.23	3.48	<0.001
		30	1.36	3.48	<0.001
	40 (55)	10	1.36	1.98	0.131
		20	0.94	1.98	<0.001
		30	1.08	1.98	0.025

UEC: urine egg count; UFM: urine filtration microscopy; FC: Fluke Catcher

**Fig 5:**
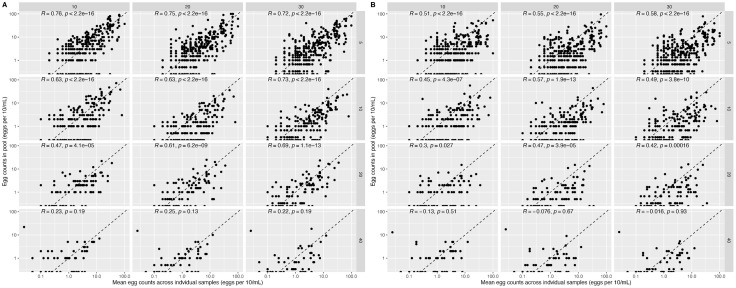
The agreement in egg counts of pooled urine samples for different pool sizes, volume of urine and diagnostic methods. The scatter plots represent the agreement in egg counts of pooled urine samples (pools of 5, 10, 20 and 40) and the corresponding mean egg counts per 10 mL of individual urine samples when 10, 20 and 30 mL of urine is examined with urine filtration microscopy (**Panel A**) and Fluke Catcher (**Panel B**). ‘*R*’ represents Pearson’s’ correlation coefficient, ‘*p*’ the corresponding *p*-value. The dashed line represents the line of equivalence.

There was a significant positive correlation between the mean UEC of pools of 5, 10, or 20 samples and the mean UEC of the corresponding individual urine samples making the pools for both UFM (*r*: 0.47-0.76) and FC (*r*: 0.30-0.58) regardless of the volume of urine (**Fig 5**). For pools of 40, the correlation coefficient was not significantly different from zero for all combinations of diagnostic methods and volume of urine.

## Discussion

This study assessed the impact of diagnostic method, volume of urine examined, pool size, and intensity of infection on the detection and quantification of *S. haematobium* eggs in pooled urine samples. The sensitivity of the pooled testing strategy increased significantly when UFM was deployed compared to the FC method. The estimated sensitivity of the pooled testing strategy also increased with an increase in the volume of urine examined when the mean UEC equaled 10 eggs per 10 mL of urine. Although the increase in diagnostic sensitivity as a function of increasing urine volume and intensity of infection is not unexpected and has been discussed in other settings [22,23,39], it has practical applications in how pooled strategies may be reconciled with surveillance needs in various collection and endemicity settings. The increased sensitivity of UFM for detecting heavy intensity infections, [3,4] and the reduced dilution effect when negative samples are added to the pool, due to higher *S. haematobium* egg counts in positive samples, could have enhanced the sensitivity of the pooled testing strategy as infection intensity increased. Similarly, since *S. haematobium* eggs are unevenly distributed in urine samples [3,4], using a larger volume may help minimize these variations and increase the probability of finding positive samples, making it easier to detect low-intensity infections in pooled samples. This study tested a low prevalence scenario by introducing single *S. haematobium*-positive specimens into each pool. A study using porcine fecal samples also showed a decreased infection detectability in pooled samples containing only one infected sample compared to pooled samples made by combining multiple samples with light infection, even when a highly sensitive PCR tool was used for examination [40].

The increased sensitivity of the pooled testing strategy seen when UFM is deployed is consistent with previous literature [40–43]. Such advantages may be attenuated over time with newer technologies. For instance, one study reported a higher sensitivity of the point-of-care circulating cathodic antigen cassette test for detecting *S. mansoni* infection in pooled urine samples than the Kato Katz thick smear technique [39]. The current study was the first study to apply FC for detecting *S. haematobium* eggs in pooled urine samples. The best way to apply FC in this setting is not yet clear, but its evaluation is an important step, recognizing the limitations in broadly distributing and sustaining high quality microscopy as an approach to community surveillance.

The present study also highlighted a trend of decreased sensitivity with an increase in the size of the pools despite lack of significance when adjusted for volume of urine, diagnostic test and egg count in the logistic regression model. Previous studies also reported an inverse relationship between the sensitivity of a pooled testing strategy and the number of individual samples pooled [14,27]. The amount of urine contributed by the individuals to the pools decreases as a function of the size of the pool. Hence, the probability of the transfer of eggs from the individual samples to the pooled samples may decrease as the pool size increases, particularly when the infection intensity is low. In addition, as the pools were made by mixing one positive individual sample and the remaining negative samples, the dilution effect in pooling the samples will be more pronounced as the size of the pool increases. This can result in false negatives, reducing overall sensitivity. If a pool is too large and the initial test fails to detect a weak positive due to dilution, that individual may not be identified in follow-up testing. However, the distribution of sourcing egg burden was not even among the pools. This lack of continuity in egg density across specimens assessed could have introduced a differential selection bias in the estimated sensitivity across diagnostic modalities.

Moreover, there was a strong positive correlation between the UECs obtained by examining pooled samples and the mean UEC of the corresponding individual samples making the pools, suggesting that an optimization approach exists to minimize the consequence of higher pool size and approach greater than minimal pool size in practice. The average UECs of the pools of 5 samples per 10 mL urine, 20 samples per 20 mL urine, and 40 samples per 20 mL urine were also comparable to the mean egg counts of the corresponding individual samples making the pools. Previous studies also have reported discernible analytic linkages between estimates of the intensity of soil-transmitted helminth infections [21,44,45] and *S. haematobium* [22,23] infections by pooled and individual testing strategies.

The present study generally reinforced the value of high-quality microscopy and influence of urine volume and source urine egg burden. Additional studies are needed that evaluate the application of such a sampling and pooling strategy on the detection of *S. haematobium* in varying scenarios of endemicity. Microsimulation studies could be used as a complement when impractical to field test the scenarios (e.g., level of endemicity, program phase, and sampling efforts) [46]. Although UFM was used to classify individual samples for pool construction, this step did not influence the diagnostic comparison, as both UFM and FC were applied independently to the same pooled specimens. Any individual-level misclassification would be non-differential and would likely bias results toward the null rather than favor either method.

Another strength of this study is the use of experimentally diluted/standardized egg concentrations to evaluate pooled testing performance under controlled conditions. The controlled single-positive design was used as a necessary first step to establish performance characteristics. By recreating low-intensity infections through dilution, we were able to isolate the effects of pool size, urine volume, and diagnostic method on sensitivity and make direct comparisons across testing strategies. Relying solely on naturally occurring infection intensities would have introduced substantial variability and limited comparability between pools, particularly given the relatively small number of heavy-intensity infections in our field samples. Nevertheless, because experimental dilution may not fully capture the biological heterogeneity of naturally infected urine, we complemented these analyses with stratified evaluations using observed field infection intensities to confirm real-world applicability (Fig 3). Future studies should further validate pooled testing strategies under entirely field-based conditions in which samples are pooled randomly regardless of infection status to evaluate the operational performance and real-world applicability of the optimized pooling strategy.

Moreover, comparing the observed egg count distributions with theoretical Poisson expectations could further inform mixing efficiency and support simulation-based optimization of pooling strategies. Although this formal modeling was beyond the scope of the present study, all samples were thoroughly homogenized before aliquoting to minimize sedimentation and ensure random egg allocation. In addition, statistical approaches are needed that allow for the estimation of the prevalence of *S. haematobium* infection in a population based upon the results of the pooled examination. While some statistical methods have been described for soil-transmitted helminths, *S. mansoni,* and different infections in vector populations, these approaches need validation for *S. haematobium* [46–48]. Moreover, there is a lack of time and other cost-benefit analyses to delineate when pooling urine samples during a large-scale epidemiological survey of *S. haematobium* infection is indeed cost-saving. This is particularly relevant for the FC, which would require more time, personnel effort, and water for testing compared to conventional UFM, potentially limiting its feasibility for field deployment in resource-limited settings. This will support program managers and healthcare decision-makers in creating the most economical survey for rapidly monitoring MDA initiatives targeted at *S. haematobium* infection control. Additionally, samples with known positive and negative results for *S. haematobium* eggs were mixed using a hierarchical strategy. The sensitivity of the UFM or FC could vary when applied to urine samples that are randomly mixed without checking for the presence of *S. haematobium* eggs. Furthermore, alternative pooling strategies, such as the array technique, may provide better sensitivity in detecting infections compared to the hierarchical approach. Additionally, the use of more sensitive molecular techniques could improve the likelihood of detecting light infections in pooled samples. Future studies could employ highly sensitive molecular diagnostic tools to analyze randomly pooled samples using both cascaded and array strategies. These studies could also explore the optimal pool sizes that minimize the expected number of tests for both array and hierarchical pooled urine testing while maintaining reliable sensitivity. Such pool sizes could then be utilized in large-scale epidemiological surveys in endemic regions. Moreover, although urine samples were also collected one month after praziquantel treatment; the number of positive samples available for pooling after treatment was relatively small. This limited our ability to perform a separate, powered analysis of post-treatment pooling performance. Pre- and post-treatment data were therefore combined, with infection intensity included in the models to account for these differences. Future studies with larger post-treatment sample sizes will be important to specifically assess the performance of pooled urine testing strategies in low-intensity, post-treatment settings and to further refine optimal pooling volumes under these conditions.

## Conclusions

In conclusion, the diagnostic sensitivity of a pooled sample examination strategy is influenced by the intricate interaction between pool size, urine volume examined, urine egg count, and the diagnostic method used. The sensitivity of the pooled testing strategy may increase with larger urine volumes, higher infection intensity, and when UFM (rather than FC) is used for testing. However, sensitivity could decrease as the pool size increases. Choosing a pooling strategy for diagnosing *S. haematobium* infection should consider the pool size, urine volume, urine egg count and diagnostic method.

## Supporting information

S1 Fig Distribution of *S. haematobium* egg counts (log eggs/10 mL urine) among study participants, stratified by study region. (TIF)

S1 Table Factors associated with the prevalence and intensity of *S. haematobium* infection from a logistic and Zero-inflated negative binomial regression models. (DOCX)

S2 Table Zero-inflated negative binomial regression of pooled egg counts. (DOCX)

S1 Data Complete anonymized dataset underlying the results reported in this manuscript. (XLSX)
