# Supplementary figures and images for: Factors affecting detection and quantification of Schistosoma haematobium eggs in pooled urine samples
Source: PLoS Negl Trop Dis. 2026 Jun 1;20(6):e0014407. doi: 10.1371/journal.pntd.0014407 (PMC13245858; doi:10.1371/journal.pntd.0014407)

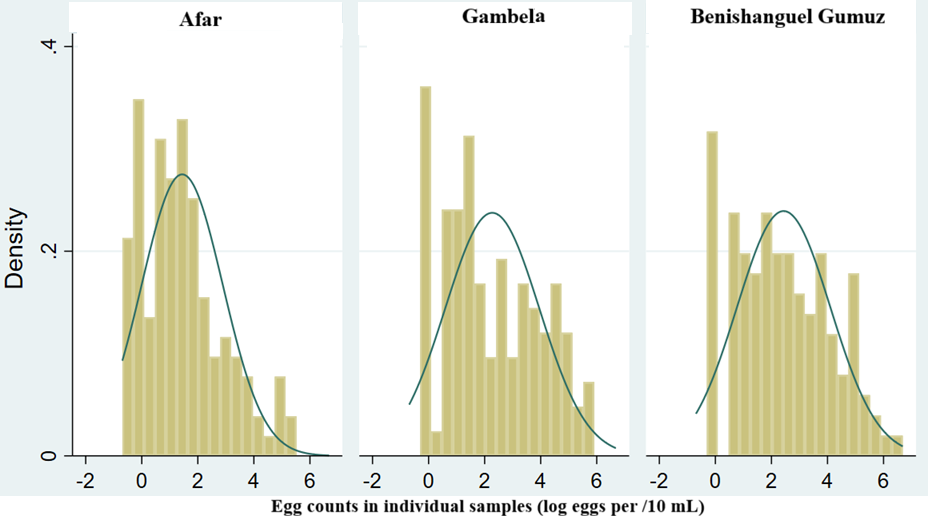

Supplement: S1 Fig — (TIF) [file pntd.0014407.s001.tif]
