# Supplementary material for: Factors affecting detection and quantification of Schistosoma haematobium eggs in pooled urine samples
Source: PLoS Negl Trop Dis. 2026 Jun 1;20(6):e0014407. doi: 10.1371/journal.pntd.0014407 (PMC13245858; doi:10.1371/journal.pntd.0014407)
Supplement: S1 Table — (DOCX) [file pntd.0014407.s002.docx]

S1 Table. Factors associated with the prevalence and intensity of *S. haematobium* infection from a logistic and Zero-inflated negative binomial regression models

|  | Logistic model (OR, 95% CI, p-value) | ZINB model (IRR, 95% CI, p-value) |
| --- | --- | --- |
| Age | 1.06 (1.02 – 1.10, 0.002) | 0.97 (0.88 – 1.07, 0.585) |
| Gender | 1.18 (0.95 – 1.47, 0.140) | 0.91 (0.56–1.47, 0.691) |
| Village | 1.02 (0.99 – 1.05, 0.180) | 1.09 (1.01 – 1.17, 0.025) |

ZINB: Zero-inflated negative binomial regression model; IRR: Incidence Rate Ratio: OR: Odds Ratio

| **Volume=30ml** |  |  |
| --- | --- | --- |
| Mean log of UEC (eggs per 10 mL of urine) | 1.05 (1.04–1.07, <0.001) | 0.13 (0.02–0.57, 0.008) |
| Pool size | 0.98 (0.97–0.99, <0.001) | 1.01 (0.961–1.06, 0.717) |
| UFM *vs.* Fluke Cather | 1.29 (1.13–1.46, <0.001) | 0.33 (0.20–0.56, <0.001) |
| Mean log of UEC x pool size | 1.002 (1.000–1.003, 0.023) | 0.77 (0.62–0.97, 0.024) |
