# Supplementary material for: Factors affecting detection and quantification of Schistosoma haematobium eggs in pooled urine samples
Source: PLoS Negl Trop Dis. 2026 Jun 1;20(6):e0014407. doi: 10.1371/journal.pntd.0014407 (PMC13245858; doi:10.1371/journal.pntd.0014407)
Supplement: S2 Table — (DOCX) [file pntd.0014407.s003.docx]

S2 Table. Zero-inflated negative binomial regression of pooled egg counts

| **Volume=10ml** | **Count model (IRR, 95% CI, p-value)** |
| --- | --- |
| Mean log of UEC (eggs per 10 mL of urine) | 1.06 (1.05–1.07, <0.001) |
| Pool size | 0.98 (0.97–0.99, <0.001) |
| UFM *vs.* Fluke Cather | 1.49 (1.30 – 1.70, <0.001) |
| Mean log of UEC x pool size | 1.001 (0.999 – 1.002, 0.395) |
| **Volume=20ml** |  |
| Mean log of UEC (eggs per 10 mL of urine) | 1.06 (1.05–1.07, <0.001) |
| Pool size | 0.98 (0.97–0.99, <0.001) |
| UFM *vs.* Fluke Cather | 1.39 (1.22–1.58, <0.001) |
| Mean log of UEC x pool size | 1.001 (0.999–1.002, 0.326) |
| **Volume=30ml** |  |
| Mean log of UEC (eggs per 10 mL of urine) | 1.05 (1.04–1.07, <0.001) |
| Pool size | 0.98 (0.97–0.99, <0.001) |
| UFM *vs.* Fluke Cather | 1.29 (1.13–1.46, <0.001) |
| Mean log of UEC x pool size | 1.002 (1.00–1.003, 0.023) |
| IRR: incidence rate ratios |  |
