# Supplementary material for: Factors affecting detection and quantification of Schistosoma haematobium eggs in pooled urine samples
Source: PLoS Negl Trop Dis. 2026 Jun 1;20(6):e0014407. doi: 10.1371/journal.pntd.0014407 (PMC13245858; doi:10.1371/journal.pntd.0014407)
Supplement: Attachment — Submitted filename: Response to Reviewers Comments.docx [file pntd.0014407.s005.docx]

**Dear Drs** **Bradbury and Tritten**

We would like to thank you for your comments and handling the submission and review process. We also thank very much the reviewers for their careful and thorough review and constructive comments, which have helped to improve the manuscript. We have made changes to the manuscript based on editors’ and reviewers' suggestions and described these changes in the below paragraphs. We hope that you will find our responses acceptable, and we look forward to your decision.

**Editors**

1) Please ensure that the CRediT author contributions listed for every co-author are completed accurately and in full.
At this stage, the following Authors/Authors require contributions: Abraham Degarege, Bruno Levecke, Christopher R Bilder, David M Brett-Major, Abebe Animut, Yohannes D Negash, M. Jana Broadhurst, Tzeyu D Michaud, and Berhanu Erko. Please ensure that the full contributions of each author are acknowledged in the "Add/Edit/Remove Authors" section of our submission form.
The list of CRediT author contributions may be found here: [https://journals.plos.org/plosntds/s/authorship#loc-author-contributions](https://nam12.safelinks.protection.outlook.com/?url=https%3A%2F%2Furldefense.com%2Fv3%2F__https%3A%2Fjournals.plos.org%2Fplosntds%2Fs%2Fauthorship*loc-author-contributions__%3BIw!!PvXuogZ4sRB2p-tU!GLkVlc3y6t8eKh-LE6g8vY0SJh2xMPz4Hxn8__bdtV7FqJ7fPZ4xi111MwSym_V5qO2s3DEzfwmcL-xcdqqYrlHlsQ%24&data=05%7C02%7Cabraham.mengist%40unmc.edu%7C6a5153661cb44dcdf2e708de6fe047ea%7C84a28940b46441c3ba3bb4fa6665bc05%7C0%7C0%7C639071206266256366%7CUnknown%7CTWFpbGZsb3d8eyJFbXB0eU1hcGkiOnRydWUsIlYiOiIwLjAuMDAwMCIsIlAiOiJXaW4zMiIsIkFOIjoiTWFpbCIsIldUIjoyfQ%3D%3D%7C0%7C%7C%7C&sdata=iP46mHgve9THx%2FEEyGg5Zm3btgaaG8E7G7mKGAFpdmg%3D&reserved=0)

**Response:** We have completed the author contribution information for each author in the “Add/Edit/Remove Authors” section of the online submission system.

2) Please upload all main figures as separate Figure files in .tif or .eps format. For more information about how to convert and format your figure files please see our guidelines: 
[https://journals.plos.org/plosntds/s/figures](https://nam12.safelinks.protection.outlook.com/?url=https%3A%2F%2Furldefense.com%2Fv3%2F__https%3A%2Fjournals.plos.org%2Fplosntds%2Fs%2Ffigures__%3B!!PvXuogZ4sRB2p-tU!GLkVlc3y6t8eKh-LE6g8vY0SJh2xMPz4Hxn8__bdtV7FqJ7fPZ4xi111MwSym_V5qO2s3DEzfwmcL-xcdqoZnrHEWA%24&data=05%7C02%7Cabraham.mengist%40unmc.edu%7C6a5153661cb44dcdf2e708de6fe047ea%7C84a28940b46441c3ba3bb4fa6665bc05%7C0%7C0%7C639071206266304172%7CUnknown%7CTWFpbGZsb3d8eyJFbXB0eU1hcGkiOnRydWUsIlYiOiIwLjAuMDAwMCIsIlAiOiJXaW4zMiIsIkFOIjoiTWFpbCIsIldUIjoyfQ%3D%3D%7C0%7C%7C%7C&sdata=phBpvhF03Cbh30N8pnshk0UFRz583zjYTU4wc4F1HUY%3D&reserved=0)

**Response:** All figures have been uploaded as separate files in .tif format, as required by the journal submission guidelines.

3) Some material included in your submission may be copyrighted. According to PLOSu2019s copyright policy, authors who use figures or other material (e.g., graphics, clipart, maps) from another author or copyright holder must demonstrate or obtain permission to publish this material under the Creative Commons Attribution 4.0 International (CC BY 4.0) License used by PLOS journals. Please closely review the details of PLOSu2019s copyright requirements here: PLOS Licenses and Copyright. If you need to request permissions from a copyright holder, you may use PLOS's Copyright Content Permission form.
Please respond directly to this email and provide any known details concerning your material's license terms and permissions required for reuse, even if you have not yet obtained copyright permissions or are unsure of your material's copyright compatibility. Once you have responded and addressed all other outstanding technical requirements, you may resubmit your manuscript within Editorial Manager. 
Potential Copyright Issues:
i) Figure 1. Please confirm whether you drew the images / clip-art within the figure panels by hand. If you did not draw the images, please provide (a) a link to the source of the images or icons and their license / terms of use; or (b) written permission from the copyright holder to publish the images or icons under our CC BY 4.0 license. Alternatively, you may replace the images with open source alternatives. See these open source resources you may use to replace images / clip-art:
- [https://commons.wikimedia.org](https://nam12.safelinks.protection.outlook.com/?url=https%3A%2F%2Furldefense.com%2Fv3%2F__https%3A%2Fcommons.wikimedia.org%2F__%3B!!PvXuogZ4sRB2p-tU!GLkVlc3y6t8eKh-LE6g8vY0SJh2xMPz4Hxn8__bdtV7FqJ7fPZ4xi111MwSym_V5qO2s3DEzfwmcL-xcdqp0dgkRVQ%24&data=05%7C02%7Cabraham.mengist%40unmc.edu%7C6a5153661cb44dcdf2e708de6fe047ea%7C84a28940b46441c3ba3bb4fa6665bc05%7C0%7C0%7C639071206266371876%7CUnknown%7CTWFpbGZsb3d8eyJFbXB0eU1hcGkiOnRydWUsIlYiOiIwLjAuMDAwMCIsIlAiOiJXaW4zMiIsIkFOIjoiTWFpbCIsIldUIjoyfQ%3D%3D%7C0%7C%7C%7C&sdata=FO%2Ff57OBfCR4%2Bt8yO3QSx40B7jj6EKD7kdY2srcnGLg%3D&reserved=0)
- [https://openclipart.org/](https://nam12.safelinks.protection.outlook.com/?url=https%3A%2F%2Furldefense.com%2Fv3%2F__https%3A%2Fopenclipart.org%2F__%3B!!PvXuogZ4sRB2p-tU!GLkVlc3y6t8eKh-LE6g8vY0SJh2xMPz4Hxn8__bdtV7FqJ7fPZ4xi111MwSym_V5qO2s3DEzfwmcL-xcdqpoq6mV5g%24&data=05%7C02%7Cabraham.mengist%40unmc.edu%7C6a5153661cb44dcdf2e708de6fe047ea%7C84a28940b46441c3ba3bb4fa6665bc05%7C0%7C0%7C639071206266422560%7CUnknown%7CTWFpbGZsb3d8eyJFbXB0eU1hcGkiOnRydWUsIlYiOiIwLjAuMDAwMCIsIlAiOiJXaW4zMiIsIkFOIjoiTWFpbCIsIldUIjoyfQ%3D%3D%7C0%7C%7C%7C&sdata=%2B1cjmf6XLyxKQRzXaYeP52rH3X2Kz%2BR9yOowIUS41lM%3D&reserved=0).

**Response**: Figure 1 was created by the authors using BioRender ( <https://www.biorender.com/>).

ii) Figure S1. Please (a) provide a direct link to the base layer of the map (i.e., the country or region border shape) and ensure this is also included in the figure legend; and (b) provide a link to the terms of use / license information for the base layer image or shapefile. We cannot publish proprietary or copyrighted maps (e.g. Google Maps, Mapquest) and the terms of use for your map base layer must be compatible with our CC BY 4.0 license.
Note: if you created the map in a software program like R or ArcGIS, please locate and indicate the source of the basemap shapefile onto which data has been plotted.
If your map was obtained from a copyrighted source please amend the figure so that the base map used is from an openly available source. Alternatively, please provide explicit written permission from the copyright holder granting you the right to publish the material under our CC BY 4.0 license.
If you are unsure whether you can use a map or not, please do reach out and we will be able to help you. The following websites are good examples of where you can source open access or public domain maps:
* U.S. Geological Survey (USGS) - All maps are in the public domain. ([http://www.usgs.gov](https://nam12.safelinks.protection.outlook.com/?url=https%3A%2F%2Furldefense.com%2Fv3%2F__http%3A%2Fwww.usgs.gov%2F__%3B!!PvXuogZ4sRB2p-tU!GLkVlc3y6t8eKh-LE6g8vY0SJh2xMPz4Hxn8__bdtV7FqJ7fPZ4xi111MwSym_V5qO2s3DEzfwmcL-xcdqq18l3-tg%24&data=05%7C02%7Cabraham.mengist%40unmc.edu%7C6a5153661cb44dcdf2e708de6fe047ea%7C84a28940b46441c3ba3bb4fa6665bc05%7C0%7C0%7C639071206266492443%7CUnknown%7CTWFpbGZsb3d8eyJFbXB0eU1hcGkiOnRydWUsIlYiOiIwLjAuMDAwMCIsIlAiOiJXaW4zMiIsIkFOIjoiTWFpbCIsIldUIjoyfQ%3D%3D%7C0%7C%7C%7C&sdata=3dHee0NG73%2BHwmurW0O7YspnDvOel9ZwDaeviXzf3WI%3D&reserved=0))
* PlaniGlobe - All maps are published under a Creative Commons license so please cite u201cPlaniGlobe, [http://www.planiglobe.com](https://nam12.safelinks.protection.outlook.com/?url=https%3A%2F%2Furldefense.com%2Fv3%2F__http%3A%2Fwww.planiglobe.com%2F__%3B!!PvXuogZ4sRB2p-tU!GLkVlc3y6t8eKh-LE6g8vY0SJh2xMPz4Hxn8__bdtV7FqJ7fPZ4xi111MwSym_V5qO2s3DEzfwmcL-xcdqpmQNYioQ%24&data=05%7C02%7Cabraham.mengist%40unmc.edu%7C6a5153661cb44dcdf2e708de6fe047ea%7C84a28940b46441c3ba3bb4fa6665bc05%7C0%7C0%7C639071206266589277%7CUnknown%7CTWFpbGZsb3d8eyJFbXB0eU1hcGkiOnRydWUsIlYiOiIwLjAuMDAwMCIsIlAiOiJXaW4zMiIsIkFOIjoiTWFpbCIsIldUIjoyfQ%3D%3D%7C0%7C%7C%7C&sdata=eTJ3qCPkt4Ln8qlIDJdpsejQUORxOHd4iOBeuVXpzyY%3D&reserved=0), CC BY 2.0u201d in the image credit after the caption. ([http://www.planiglobe.com/?lang=enl](https://nam12.safelinks.protection.outlook.com/?url=https%3A%2F%2Furldefense.com%2Fv3%2F__http%3A%2Fwww.planiglobe.com%2F%3Flang%3Denl__%3B!!PvXuogZ4sRB2p-tU!GLkVlc3y6t8eKh-LE6g8vY0SJh2xMPz4Hxn8__bdtV7FqJ7fPZ4xi111MwSym_V5qO2s3DEzfwmcL-xcdqoX7moUAw%24&data=05%7C02%7Cabraham.mengist%40unmc.edu%7C6a5153661cb44dcdf2e708de6fe047ea%7C84a28940b46441c3ba3bb4fa6665bc05%7C0%7C0%7C639071206266642856%7CUnknown%7CTWFpbGZsb3d8eyJFbXB0eU1hcGkiOnRydWUsIlYiOiIwLjAuMDAwMCIsIlAiOiJXaW4zMiIsIkFOIjoiTWFpbCIsIldUIjoyfQ%3D%3D%7C0%7C%7C%7C&sdata=rF4PrBz05dqJhoqM3ZTvzLCYgz0i0a22ufrNth%2FmQq8%3D&reserved=0))
* Natural Earth - All maps are public domain. ([http://www.naturalearthdata.com/about/terms-of-use/](https://nam12.safelinks.protection.outlook.com/?url=https%3A%2F%2Furldefense.com%2Fv3%2F__http%3A%2Fwww.naturalearthdata.com%2Fabout%2Fterms-of-use%2F__%3B!!PvXuogZ4sRB2p-tU!GLkVlc3y6t8eKh-LE6g8vY0SJh2xMPz4Hxn8__bdtV7FqJ7fPZ4xi111MwSym_V5qO2s3DEzfwmcL-xcdqowL2ii8g%24&data=05%7C02%7Cabraham.mengist%40unmc.edu%7C6a5153661cb44dcdf2e708de6fe047ea%7C84a28940b46441c3ba3bb4fa6665bc05%7C0%7C0%7C639071206266694125%7CUnknown%7CTWFpbGZsb3d8eyJFbXB0eU1hcGkiOnRydWUsIlYiOiIwLjAuMDAwMCIsIlAiOiJXaW4zMiIsIkFOIjoiTWFpbCIsIldUIjoyfQ%3D%3D%7C0%7C%7C%7C&sdata=H4khkwRTZtUnWVXQivxouyo0OOkKffLbfy%2BAEXGBQoU%3D&reserved=0)).

**Response**: The information in S1 Figure is supplementary; therefore, we have removed the figure from the main manuscript.

4) Kindly revise your competing statement in the online submission form to align with the journal's style guidelines: 'The authors declare that there are no competing interests.'

**Response**: We have revised the competing interests statement in both the manuscript and the online submission to read: “The authors declare that there are no competing interests.”

**Reviewer #1:**

The authors are dealing with an important topic which should have great impact in the era of schistosomiasis elimination and in the context of limited financial resources. Please find below comments and suggestions for improvement that the authors could trust in.
**Methods**
1. This study would have yielded more interesting results if the authors had not created conditions of low infection intensity by diluting the samples, but had instead considered the low, moderate, and heavy infections from their field sampling. This is especially relevant given the large sample size. Can the authors explain their choice to recreate conditions of low infection intensity?

**Response**: We thank the reviewer for this insightful comment and for highlighting the importance of evaluating pooled testing performance across naturally occurring infection intensities. Our primary objective in this study was to systematically assess how pool size, urine volume, infection intensity, and diagnostic modality influence the sensitivity of *S. haematobium* detection under controlled conditions in order to optimize pooled testing parameters.

To achieve this, we experimentally recreated lower infection intensities through dilution of positive urine samples. This approach allowed us to standardize egg concentrations and generate consistent, reproducible intensity levels across multiple pool sizes and testing conditions. Using only naturally occurring low-, moderate-, and heavy-intensity field samples would have limited our ability to control egg distribution within pools and to make direct comparisons across experimental configurations. In addition, despite the overall large sample size, the number of individuals with heavy-intensity infections was relatively small (n = 64), which restricted statistical power for stratified analyses across different urine volumes, diagnostic methods, and pool sizes.

Nevertheless, we agree that evaluating performance across field-derived infection categories is important for assessing real-world applicability. To address this, we also present analyses stratified by observed infection intensity from field samples (Figure 3), which demonstrate similar trends in sensitivity. We have clarified this rationale and the complementary role of both approaches in the revised manuscript **(See discussion page 22 & 23).**

The added text reads as “Another strength of this study is the use of experimentally diluted/standardized egg concentrations to evaluate pooled testing performance under controlled conditions. The controlled single-positive design was used as a necessary first step to establish performance characteristics. By recreating low-intensity infections through dilution, we were able to isolate the effects of pool size, urine volume, and diagnostic method on sensitivity and make direct comparisons across testing strategies. Relying solely on naturally occurring infection intensities would have introduced substantial variability and limited comparability between pools, particularly given the relatively small number of heavy-intensity infections in our field samples. Nevertheless, because experimental dilution may not fully capture the biological heterogeneity of naturally infected urine, we complemented these analyses with stratified evaluations using observed field infection intensities to confirm real-world applicability (Fig 3). Future studies should further validate pooled testing strategies under entirely field-based conditions in which samples are pooled randomly regardless of infection status to evaluate the operational performance and real-world applicability of the optimized pooling strategy.”

2. L17 – L22: When did you use the FC?

**Response**: Thank you. FC was employed for sample examination in the lab (not in the field). We have explained this in the methods. The revised text reads as “Urine samples with *S. haematobium* eggs detected were pooled with negative samples and examined using UFM and FC at the Medical Parasitology Laboratory of Aklilu Lemma Institute of Pathobiology of Addis Ababa University.” **(see methods: study design)**

3. Please combine both “Study rea” and “Study population” and named “Study area and population”

**Response**: Thank you. We have combined the text under the “Study area” and “Study population” into one under the heading “Study population and study area” (see methods).

4. L149: Please remove this “(=995+995+498 +100).” **Response**: Thank you. We have removed it **(see methods: sample size).**

5. L144-L145: Not clear how the following numbers have been reaching “we needed to screen a minimum of 995 children in Afar, 995 children in Benishangul-Gumuz, and 498 in Gambella” Please, could describe more.

**Response**: Thank you. We have clarified how the sample sizes for each region was estimated. The revised text on sample size reads as follows **(see methods: sample size)**:

“The sample size for each study region was estimated using published sample size tables from a review article that summarize the minimum number of samples required for sensitivity and specificity analyses [31]. These estimates were calculated using Power Analysis and Sample Size (PASS) software, incorporating specified confidence interval levels, statistical power, and disease prevalence assumptions [32]. We previously found a 51.7% sensitivity of the hierarchical pooling technique for detecting S. haematobium infection using UFM with 10 mL urine (Ho) [23]. In order to test the hypothesis that the sensitivity of the pooled testing is at least 60% (Ha) when the volume of urine examined is >10 mL with a power of 80% (type II error of 20%) and type I error level of 5%, we needed to screen a minimum of 995 children in Afar and 995 children in Benishangul-Gumuz where the prevalence of S. haematobium infection was estimated to be 20%, and 498 in Gambella where the prevalence of S. haematobium infection was estimated to be 40% [22,23,28-30]. Due to the low prevalence rate in some villages in the Afar region, we proposed to collect samples from an additional 100 (10% of 995) children in Afar to ensure the collection of an adequate number of positive samples to assess sensitivity. The total sample size estimated for this study was 2,588.”

6. Which formular has been used to assess the sample size?

**Response**: Thank you. Please see our response above to comment no # 5.

7. L153-L155 “Then, the …. whose parents approved their participation”. When and how the consent from the parents have been collected.

**Response**: Thank you. We have added details on the consent and assent process both on the data collection and ethics statement.

The added text under the heading “urine sample collection and processing” reads as “Then, the study team recruited children who agreed to participate, and whose parents or guardians approved their participation (see ethics statement). Eligible children were recruited using consecutive (convenience) sampling from predefined community collection points, including nearby health posts, schools, and open field stations within the villages. Consent discussion was conducted privately in nearby health posts, schools, or designated open fields within the villages, where the study objectives, procedures, potential benefits, and risks were explained. Following consent and assent, participating children were asked to provide at least 80 mL in labeled 200 mL plastic containers between 10:00 am and 3:00 pm.” **(see methods: urine sample collection and processing).**

The revised text under the heading “ethical considerations” reads as follows: “Written informed consent without a signature was obtained from parents or guardians of children who assent to participate in the study. As the study population was members of a distinct cultural group or community in which signing forms is not the norm, the institutional review board approved a waiver of signed consent for this study. Accordingly, parents or legal guardians received written information describing the study objectives, procedures, risks, and benefits. Verbal informed consent was obtained in lieu of written signatures, with a study team member serving as witness to confirm that the information was explained, questions were answered, and participation was voluntary.” **(see methods: ethical consideration).**

8. L155 – L157: “Between 10:00 am and 3:00 pm, children who came to the nearby health posts, schools, or open fields, were asked to bring a urine sample of at least in labeled 200 mL plastic containers” Please, which kind of sampling method was used? This part raises some concerns about how consent has been obtained from children’s parents.

**Response**: Thank you for this important comment. Eligible children were recruited using consecutive (convenience) sampling from predefined community collection points, including nearby health posts, schools, and open field stations within the villages. All children were recruited only after parental/guardian permission and child assent were obtained. We have revised the Methods section to clarify both the sampling approach and the consent procedures **(****Please see:** **Methods:** “**Urine sample collection and processing” and “ethical consideration”**).

9. L161 “UFM results were shared with the children and their parents at the collection site” How this has been done since available cases nearby health posts, schools or open fields were targeted?

**Response**: Thank you for the comment. UFM results were shared confidentially with participating children and their parents or guardians at the sample collection sites, with support from local health personnel or nurses and school staff or village officials when needed. We have added this text to the method section of the manuscript **(Please see:** **Methods:** “**Urine sample collection and processing”**).

10. L166 “We first re-tested 10 mL of the formalin-fixed urine samples from each child using UFM.” When has this been done after the fieldwork?

**Response**: Thank you. Yes, 10 ml urine sample was tested once again deploying UFM in the Medical Parasitology Laboratory of Aklilu Lemma Institute of Pathobiology of Addis Ababa University. We have updated the text in the method to make this clear. The revised text reads as “We first re-tested 10 mL of the formalin-fixed urine samples from each child using UFM after the field data collection.” **(Please see:** **Methods:** “**Urine sample collection and processing”**).

11. Why has formalin been used?

**Response**: Formalin was added to urine samples to preserve *Schistosoma haematobium* egg morphology and prevent sample degradation during storage and transport from the field to the laboratory. This was particularly important because samples could not always be processed immediately after collection under field conditions. The use of formalin ensured reliable detection and enumeration of eggs by urine filtration microscopy.

12. L173 “In each group, one positive sample was included, while the remaining samples were negative.” How has this sample been selected? What was the infection intensity of the selected one? What is the rationale behind this design of putting only one negative among the positives? It seems that this is far from the real world. Why not pool regardless of the infection status and record the type of samples pooled and consider the sample characteristics during the analysis?

**Response**: Thank you for this important comment. “*The positive sample included in each pool was selected at random from the set of known positive specimens and was not chosen based on infection intensity*.” Consequently, pools represented a range of egg burdens across experiments. We have clarified this point in the revised Methods section **(Please see: Methods: “Urine sample collection and processing”).**

Our decision to include a single positive specimen per pool was intentional and reflects the experimental objective of this study. Specifically, this was a controlled optimization study designed to evaluate the analytical performance of pooled testing under defined conditions and to determine the detection limits of the assay. By constructing pools with exactly one positive sample diluted among negatives, we were able to systematically assess how pool size, urine volume, diagnostic modality, and infection intensity influence sensitivity. This design isolates the dilution effect and provides a conservative estimate of detection performance, which is essential for identifying the maximum pool size at which infections of varying intensities remain detectable.

We agree that field conditions typically involve pools with unknown and potentially multiple positives. However, pooling irrespective of infection status would introduce heterogeneity that makes it difficult to quantify assay detection thresholds and optimize pooling parameters. Therefore, the controlled single-positive design was used as a necessary first step to establish performance characteristics.

Importantly, we are conducting a follow-up field-based validation study in which samples are pooled randomly regardless of infection status to evaluate the operational performance and real-world applicability of the optimized pooling strategy. We have added text to the manuscript to clarify this rationale and to better distinguish between the experimental optimization phase and the planned field implementation **(see discussion page 22 & 23).**

13. L234 – L236 “As the study population was members… institutional review board approved a waiver of signed consent for this study.” Then you clearly mentioned that above to help understanding.

**Response**: Thank you. We have explained that. Please see our response to comment #7.

14. L254: “Figure 2. The prevalence and mean UEC” Which mean has been used? Arithmetic one or the geometric one.

**Response**: Thank you. The values in Fig 2 are arithmetic mean. We have updated the legend for figure 2 to reflect this.

**Discussion**
15. L262 “Overall, the diagnostic sensitivity tends to increase as a function of the intensity level of infection and the volume of urine examined” Not sure if any investigation is required to support this statement. This is so obvious!!!

**Response**: Thank you. We agree that the observed increase in diagnostic sensitivity with higher infection intensity and larger urine volume is biologically and methodologically expected. Greater egg burden increases the probability of egg capture within pooled samples, and examining larger urine volumes similarly increases the likelihood of detecting eggs, thereby improving test sensitivity.

To support this observation, we formally evaluated the relationship between sensitivity, infection intensity, and urine volume by stratified analysis and logistic regression. The analyses confirmed a positive association between both higher intensity categories and larger sample volumes with diagnostic sensitivity (Fig 3 & 4; Table 1). We have also discussed the implication of the results in deploying pooled testing strategies for surveillance needs in various collection and endemicity settings (see discussion; page 20).

16. L350: “Overall, the diagnostic sensitivity tends to increase as a function of the intensity level of infection and the volume of urine examined” This assumption has never been highlighted in the ease the understanding of the overall work.

**Response**: We thank the reviewer for this observation. We agree that the relationship between diagnostic sensitivity, infection intensity, and urine volume is an important point that underpins the interpretation of our results. Although this relationship is biologically intuitive, we recognize that it was not explicitly highlighted in the original manuscript.

To improve clarity and ease the reader’s understanding, we have now revised the results and discussion section to explicitly describe that higher infection intensity and larger urine volumes are associated with increased likelihood of egg detection in pooled urine samples. We also emphasize that this relationship provides context for interpreting the observed sensitivity patterns across different pooling strategies **(Please see added results under the heading “ Factors affecting the diagnostic sensitivity” and Discussion sections (pages 15 and 20)**.

**Reviewer #2:**

I have only very minor comments. This manuscript provides valuable insights into whether pooling urine samples can facilitate population-based monitoring of Schistosoma haematobium infections. The text is well written, and the procedures are clearly described. The overall conclusion is highly relevant to the field.

I read the manuscript with great interest. The hypothesis is clearly stated, and the methods and procedures are well described. While I am not a statistical expert, the sample size and data analysis appear more than appropriate. The authors have an excellent track record in this field, which adds credibility to their work.

1. Line 162: children who tested positive when tested at the collection site were treated with praziquantel. Please explain what happened with the children who were in first instance negative, but tested positive when the 10mL urine examination was repeated (Line 166) just before the experimental pooling.

**Response**: Thank you for this helpful comment. Children who tested negative for S. haematobium by urine filtration microscopy (UFM) in the field, but were found positive in the laboratory, were treated during the second-round survey, which included re-examination of those treated one month earlier. We clarified this now in the methods **(please see “Urine sample collection and processing”, page 10). The added text reads “**During the second-round survey, children who tested negative for S. haematobium using UFM in the field, but were subsequently found positive in laboratory testing received treatment**.”**

2. Line 166: please indicate the time period (median and range) between urine collection and the experimental pooling.

**Response**: Thank you for this helpful comment. “Experimental pooling of urine samples was carried out in the laboratory, approximately 1–3 weeks after their collection in the field.” We have clarified this in the manuscript. **(please see “Urine sample collection and processing, page 9”).**

3. Line 398 mentions the lack of a time-benefit and cost-benefit study. I would appreciate some additional words about the practicality of using the Fluke Catcher compared to Urine Filtration Microscopy.

**Response**: Thank you for this helpful comment. We have discussed the potential challenges in deploying fluke catcher for testing *S. haematobium* in pooled urine samples in the manuscript. The added text reads as “This is particularly relevant for the FC, which would require more time, personnel effort, and water for testing compared to conventional UFM, potentially limiting its feasibility for field deployment in resource-limited settings.” **(please see discussion page # 24).**

**Reviewer #3:**

This manuscript presents comparative results of two laboratory methods applied to over 2000 urine samples from school-aged children, as well as a series of pools made up of test-positive and test-negative samples. The subject is worthy of investigation and there is a large body of data available (which it is fantastic to see being supplied as supplementary information - thanks!), but the presentation of the manuscript and obvious gaps in the analysis as well as theoretical statistical background currently limit the applicability and impact of the work. I therefore have a number of comments to address before publication.

1. The description of the pooling process does not state if the urine samples (both the individual samples and resulting pools) were mixed before aliquots were taken, but since this would be standard practice when creating pooled samples in other applications I assume this is the case. Perfect mixing would imply that the number of eggs in each aliquot would be Poisson distributed (with mean given by the product of the number of eggs in the sample and proportion of the sample taken as the aliquot), amd therefore the number of eggs in the resulting pooled sample would also be Poisson distributed (as the sum of independent Poisson processes is itself Poisson). This gives a theoretical basis for the expected distribution of eggs (based on the observed egg counts from the individual samples) that would be very useful to compare to the actual number of observed eggs, but this analysis is currently completely absent. This would greatly add to the paper: if the observed distribution closely matches the theoretical expectation, then this would support the use of computer simulation to analyse different pooling strategies. On the other hand, any departures from the theoretical expectation can be taken as evidence that the mixing process is imperfect and/or the egg recovery of the laboratory methods is biased.

**Response**: We thank the reviewer for highlighting this important methodological consideration. Prior to aliquoting, all individual urine samples and pooled samples were thoroughly homogenized by gentle inversion and agitation to ensure an even distribution of eggs, consistent with standard laboratory practice. This clarification has now been added to the Methods section. (**see “Urine sample collection and processing”**).

We also appreciate the reviewer’s suggestion regarding the theoretical Poisson framework for egg distribution. A binary response is examined much more in group testing because the ultimate goal is to determine positive or negative. This is why we focused on it. However, we found that there is overdispersion (i.e., do not meet Poisson distribution) in egg count in both the individual and pooled urine samples. Hence, we applied zero inflated negative binomial regression model to examine association of i) age, gender and village of residence with the intensity of *S. haematobium* infection (egg count in individual samples); ii) pool size, diagnostic method and egg count in individual samples with egg counts in pooled samples. We have described the analyses procedure and the results in the revised manuscript **(see below the details in our response to comment #4 below).** In addition, while a formal comparison between the observed and theoretical distributions was beyond the primary scope of the current study, we agree that such modeling could provide additional insight into pooling efficiency and mixing assumptions. We have now acknowledged this point in the Discussion as a potential direction for future work. It reads “Moreover, comparing the observed egg count distributions with theoretical Poisson expectations could further inform mixing efficiency and support simulation-based optimization of pooling strategies. Although this formal modeling was beyond the scope of the present study, all samples were thoroughly homogenized before aliquoting to minimize sedimentation and ensure random egg allocation” **(see discussion page 23)**.

2. There is a substantial body of literature on the utility (and approaches to) pooling for diagnosis, going all the way back to the 1940's. A lot of the lessons learned from this literature would be useful here, I think - or at the very least it should be acknowledged. You could usefully start with [https://doi.org/10.1016/j.cmi.2021.04.007](https://nam12.safelinks.protection.outlook.com/?url=https%3A%2F%2Fdoi.org%2F10.1016%2Fj.cmi.2021.04.007&data=05%7C02%7Cabraham.mengist%40unmc.edu%7C360053e63ef64f5f4aa208de5a785630%7C84a28940b46441c3ba3bb4fa6665bc05%7C0%7C0%7C639047670058595755%7CUnknown%7CTWFpbGZsb3d8eyJFbXB0eU1hcGkiOnRydWUsIlYiOiIwLjAuMDAwMCIsIlAiOiJXaW4zMiIsIkFOIjoiTWFpbCIsIldUIjoyfQ%3D%3D%7C0%7C%7C%7C&sdata=5ksd4n%2FlFxE3iSEI1WkMYjbXopJCtsSHrSEOsKbDhNA%3D&reserved=0) and go from there.

**Response**: Thank you for this helpful and insightful suggestion. We agree that pooled testing has a long and well-established history in diagnostic and surveillance applications, dating back to the seminal work of Dorfman and subsequent methodological developments across infectious disease screening. We appreciate the recommended reference and have now reviewed this and related literature.

The added text reads as “CPT has a very long history for a diverse set of applications. While Robert Dorfman [7] is usually given credit as the first to suggest the process, there are records of CPT being used as far back as 1915 [9]. Since then, CPT has been refined and widely used across applications including SARS-CoV-2 testing [10], blood donation screening [11], sexually transmitted infection detection [12], and animal disease testing [13]. General textbook summaries of CPT are available from a variety of sources [9,14,15] and from general overview papers [9,16,17].” **(Please see introduction: page 5).**
3. Given that you have paired samples from each laboratory method applied to each (pooled) sample, I was disappointed not to see a direct comparison. For example, a paired qq plot (perhaps separated by volume of urine) would show if the observed increased probability of observing zero eggs for one test was mirrored for other egg values - i.e. is the bias between the tests consistent, or dependent on the number of eggs in the sample?

**Response**: We thank the reviewer for this comment. Figure 5 provides pairwise scatter plots for the mean egg counts in the individual samples when compared to the mean egg counts in pools. We condition on urine volume and pool size for these plots. We can see that as the pool size increases the strength of correlation between the mean UEC of pooled samples and the mean UEC of the corresponding individual urine samples making the pools decreased. We can also see a trend of increased strength of correlation between the mean UEC of pooled samples and the mean UEC of the corresponding individual urine samples making the pools as the urine volume increased.

If your comment here was meant if a different way, please let us know.

4. The logistic regression is one possible analysis, but did the authors consider fitting an over-dispersed count distribution to the data, with pool size as a fixed effect? I would expect the over-dispersion to depend on the volume of urine used, so this should probably be a stratified analysis and the over-dispersion parameters compared. This is related to point (1) above (and could be used as an analysis method against which to compare the expected distribution). It would also be useful to fit a similar model (perhaps using a zero-inflated over-dispersed count model) to the individual data, to concurrently estimate risk factors for intensity and true prevalence of infection.

**Response**: We thank the reviewer for this important suggestion. Because egg counts are over-dispersed and highly zero-inflated, we agree that modeling the counts directly provides a more appropriate framework than dichotomizing outcomes using logistic regression. We therefore fitted zero inflated negative binomial regression models to assess factors associated with egg count in individual and pooled samples. For the egg count in pooled samples; pool size, egg count of the individual samples making the pools and diagnostic methods included as a fixed effect and urine volume incorporated as an offset to account for differing sample volumes. To assess whether dispersion varied by urine volume, we conducted stratified analyses and compared dispersion parameters across strata. For egg count in the individual samples, we included factors such as age, gender and village in the model. We have updated the analyses in the Methods and Results sections to reflect this. The added text in the methods reads as follows:

**Data management and statistical data analysis, page 11 & 12:**

“To assess factors associated with the intensity of S. haematobium infection, we fitted zero-inflated negative binomial (ZINB) regression models to account for overdispersion and excess zero egg counts. Age, gender, and village of residence were included as covariates. Stata was used to find the zero-inflated negative binomial model [36].

Egg counts from pooled urine samples were modeled using ZINB regression to account for overdispersion and excess zeros. Pool size (5,10,20,40), the mean egg count of constituent individual samples, diagnostic method (UFM vs FC) and their interaction were included as covariates to estimate factors associated with egg count/intensity in pooled urine samples. Stratified analyses by urine volume (10, 20, and 30 mL) were conducted to evaluate differences in dispersion across strata. Estimates/results from ZINB are reported as incidence rate ratios (IRRs), with 95% confidence intervals.”

**Results (page 14 & 17)**

In zero-inflated negative binomial models, there was moderate evidence for village being associated with S. haematobium infection intensity (IRR = 1.09, 95% CI: 1.01–1.17, p = 0.025), whereas there was not sufficient evidence that age and gender were associated with infection intensity (S1 Table).

Zero-inflated negative binomial regression model was fitted to account for overdispersion and excess zeros in pooled egg counts. In the negative binomial egg count, across all urine volumes (30, 20, and 10 mL), egg count or intensity of infection in pooled urine samples decreased consistently with an increase in pool size (IRR ≈ 0.98, p<0.001) but increased with an increase in mean individual egg counts making the pools (IRR range: 1.05–1.06, p<0.001) and when UFM used for the diagnosis than FC (IRR range: 1.29–1.49, p<0.001) (S2 Table).

**I also found the following more minor issues:**

5. I note that the individual samples were classified as positive/negative (for pooling) based on UFM test only - could this have in some way affected the comparison of UFM and FC? I don't think so, but it would be good to see this considered explicitly.

**Response**: Thank you for this thoughtful comment. We agree that the choice of reference method for classifying individual samples should be considered explicitly. In our study, UFM results were used solely to determine egg count in individual samples because UFM is the current field standard. Both UFM and FC were applied independently to the same pooled specimens, and egg detection and quantification were based on direct examination of the pooled samples rather than on the initial individual classifications.

Therefore, the use of UFM for individual sample classification would not be expected to systematically bias the relative performance comparison between UFM and FC in pooled samples, since neither method’s pooled-sample results depended on the prior classification. At most, any misclassification at the individual level would introduce non-differential variability in pool composition, which would tend to attenuate, rather than favor, differences between methods.

We have now clarified this rationale in the Discussion section **(Page 22)**. It reads as “Although UFM was used to classify individual samples for pool construction, this step did not influence the diagnostic comparison, as both UFM and FC were applied independently to the same pooled specimens. Any individual-level misclassification would be non-differential and would likely bias results toward the null rather than favor either method.”

6. I think that Figure 2A and 2B would be more informative as histograms or similar - then we can see the full distribution (or perhaps adding a histogram for each region, or similar, if you don't want to have too many histograms).

**Response**: Thank you. We have added histograms showing the distribution of *S. haematobium egg counts* (log eggs/10 mL urine) among study participants, stratified by study region (S2 Fig)

7. Figures 3 and 4 are far too large considering how little information they convey - and is figure 4 not just model predictions? These can be replaced with something more useful, I think.

**Response**: Thank you for this helpful suggestion. We agree that figures should be as concise and information dense as possible. Figures 3 and 4 were intentionally presented at their current size to preserve readability of the axes and stratified comparisons, which become difficult to interpret when reduced. Both figures show diagnostic performance based on the pool size, volume of urine, intensity of infection and diagnostic method to facilitate visual interpretation of effect sizes and trends that are less easily conveyed in tabular form. In particular, Figure 4 displays model-based predictions. We believe that presenting these results graphically improves accessibility and complements the regression estimates reported in the table 2. We respectfully propose retaining the current figure size to optimize clarity for readers.

8. Please correctly cite both R and the R packages (e.g. ggplot2) that you use for your analysis

Response: Thank you for this suggestion. We have now added proper citations for R (R Core Team, 2023) and relevant R packages used in the analyses, including ggplot2 (Wickham, 2016) and others as appropriate. References have been included in the Methods section and the reference list according to journal guidelines.

R software:

R Core Team. R: A language and environment for statistical computing. R Foundation for Statistical Computing, Vienna, Austria. 2023. URL: https://www.R-project.org/

ggplot2 package:

Wickham H. ggplot2: Elegant Graphics for Data Analysis. 2nd edition. Springer, 2016. ISBN 978-3-319-24277-4.

9. Although it is highly commendable to include the raw data as supplementary material, I am concerned about the information (age/sex/village) not meeting the standard required for anonymity (e.g. if there are fewer than 5 children of given age and gender in a specific village, we would typically consider this to be potentially re-identifiable personal [sensitive, as it is health-related] data). Would it not be prudent to anonymise the village names, and possibly also group the ages to ensure that all combinations of identifying features have at least 5 individuals?

**Response**: We thank the reviewer for highlighting this important point regarding participant confidentiality. We agree that the combination of age, sex, and village could potentially allow re-identification of individual participants in small strata. To address this, we have now:

1. Anonymized village identifiers in the supplementary dataset, replacing actual village names with unique codes.
2. Grouped ages into broader categories (e.g., 5–8, 9-12, 13–15 years) to ensure that all combinations of age, sex, and village include at least five individuals, thereby reducing re-identification risk.

These measures preserve the utility of the raw dataset for replication and secondary analyses while fully maintaining participant anonymity in accordance with ethical standards. The supplementary material has been updated accordingly.
