# Supplementary material for: Factors affecting detection and quantification of Schistosoma haematobium eggs in pooled urine samples
Source: PLoS Negl Trop Dis. 2026 Jun 1;20(6):e0014407. doi: 10.1371/journal.pntd.0014407 (PMC13245858; doi:10.1371/journal.pntd.0014407)
Supplement: Attachment — Submitted filename: Response to editors and reviewers comments .docx [file pntd.0014407.s006.docx]

**Dear Drs Coffeng, Tritten, Kamhawi and Brindley**

We would like to thank you for your comments and handling the submission and review process. We also thank very much the reviewers for their careful and thorough review and constructive comments, which have helped to improve the manuscript. We have made changes to the manuscript based on editors’ and reviewers' suggestions and described these changes in the below paragraphs. We hope that you will find our responses acceptable, and we look forward to your decision.

**Editor (s)**

1. Please perform a last check of the manuscript for spelling. Examples of typos are "aliqute" and "positisve".

**Response**: We thank the editor for this observation. We have carefully reviewed the entire manuscript and corrected spelling and typographical errors, including those noted (“aliqute” and “positisve”). We have also performed an additional thorough proofreading to ensure overall clarity and accuracy.

2. In addition, please consider rephrasing some of the following statements, which seem to cause confusion with some of the reviewers:

   a. LINE 123: “The aim of our study was to explore when ……. S. haematobium infections in the context of an MDA program.”

--> Reviewer #1:: What do you mean by “When” I did not find results related to that “when” in the MS

**Response**: Thank you for the comment. We have revised (removed “when”) the sentence. The revised sentence reads as “The aim of our study was to explore how pooled urine testing can be optimally implemented for monitoring S. haematobium infections in the context of an MDA program.”

b. LINE 132: “Urine samples were collected at baseline and one month after praziquantel was administered to children infected with the parasite based on urine testing results.” --> Reviewer #1: Did you not find the results related to posttreatment performance of the pooling and which volume if the best one?

**Response**: Thank you for this important observation. We agree that evaluating the post-treatment performance of pooled urine testing would provide valuable additional insight.

In this study, urine samples were indeed collected one month after praziquantel administration. However, the number of positive samples available for pooling after treatment was relatively small. As a result, the post-treatment dataset did not provide sufficient statistical power to support a separate, robust analysis of pooling performance (including comparisons across pool sizes and urine volumes).

To maximize the use of available data and maintain analytical reliability, we therefore combined pre- and post-treatment samples in the main analysis. We acknowledge this as a limitation of the study and have clarified it in the revised manuscript. The added text reads as “Moreover, although urine samples were also collected one month after praziquantel treatment; the number of positive samples available for pooling after treatment was relatively small. This limited our ability to perform a separate, powered analysis of post-treatment pooling performance. Pre- and post-treatment data were therefore combined, with infection intensity included in the models to account for these differences. Future studies with larger post-treatment sample sizes will be important to specifically assess the performance of pooled urine testing strategies in low-intensity, post-treatment settings and to further refine optimal pooling volumes under these conditions.”

c. LINE 438: Similarly, since S. haematobium eggs are unevenly distributed in urine samples [3,4], using a larger volume may help minimize these variations (i.e., dilute random fluctuations in egg concentration), making it easier to detect low-intensity infections in pooled samples.
--> Editor: Avoid "dilute", which seems to refer to literal dilution, whereas I presume the authors actually mean "reduce" or more specifically "reduce the probability of finding zero eggs"? If this is not the intended message, I'm not sure I understand.

**Response**: We thank the editor for this helpful clarification. We agree that the term “dilute” may be misleading in this context. Our intention was to indicate that examining a larger urine volume reduces the impact of random variation in egg distribution and, specifically, lowers the probability of observing zero eggs in low-intensity infections. We have revised the sentence accordingly to improve clarity and avoid any unintended implication of literal dilution.

**Reviewers' Comments:**

**Reviewer #1:  Major comment**

1. = The title: “Detection and quantification of Schistosoma haematobium eggs in pooled urine samples” Must be revised with consideration to the content of the MS.

**Response**: Thank you for the comment. We have revised the title to make it specific and align with the abstract. The revised title reads as “Factors affecting detection and quantification of *Schistosoma haematobium* eggs in pooled urine samples”

2.= In the abstract: “(=0.01, p=0.021) or pool size (=-0.02, p=0.007)”. What is front of the “=” otherwise that does not make sense.

**Response**: There was the symbol “$\hat{\beta}$” (representing regression coefficient) before the equal sign. Perhaps, the symbol “$\hat{\beta}$” might have been omitted from the PDF version of the manuscript.

3. = Lack of clear take away message in the conclusion and did not include all the methodologies and key results regarding the initial hypothesis of the current work. Which pool or volume is the good one and why? The way the authors concluded here seems to be more focused on already known findings, nothing new has been provided based on their own work.

**Response**: Thank you for the comment. We have revised the conclusion in the abstract. It reads as “The diagnostic sensitivity of pooled testing for diagnosing *S. haematobium* infection is affected by a complex interplay between the pool size, the volume of urine examined, the mean UEC and the diagnostic method. The sensitivity of the pooled testing strategy may increase with larger urine volumes, higher infection intensity, and when UFM (rather than FC) is used for testing. However, sensitivity could decrease as the pool size increases. Use of UFM, larger urine volumes and smaller to moderate pool sizes could improve detection of *S. haematobium* infection in surveillance and mass deworming programs in low-intensity settings.”

4. = L123 “The aim of our study was to explore when ……. S. haematobium infections in the context of an MDA program.” What do you mean by “When” I did not find results related to that “when” in the MS.

**Response**: Thank you for the comment. We have revised (removed “when”) the sentence. The revised sentence reads as “The aim of our study was to explore how pooled urine testing can be optimally implemented for monitoring *S. haematobium* infections in the context of an MDA program.”

5. = L132 “Urine samples were collected at baseline and one month after praziquantel was administered to children infected with the parasite based on urine testing results.” Did you not find the results related to posttreatment performance of the pooling and which volume if the best one?

**Response**: Thank you for this important observation. We agree that evaluating the post-treatment performance of pooled urine testing would provide valuable additional insight.

In this study, urine samples were indeed collected one month after praziquantel administration. However, the number of positive samples available for pooling after treatment was relatively small. As a result, the post-treatment dataset did not provide sufficient statistical power to support a separate, robust analysis of pooling performance (including comparisons across pool sizes and urine volumes).

To maximize the use of available data and maintain analytical reliability, we therefore combined pre- and post-treatment samples in the main analysis. We acknowledge this as a limitation of the study and have clarified it in the revised manuscript. The added text reads as “Moreover, although urine samples were also collected one month after praziquantel treatment; the number of positive samples available for pooling after treatment was relatively small. This limited our ability to perform a separate, powered analysis of post-treatment pooling performance. Pre- and post-treatment data were therefore combined, with infection intensity included in the models to account for these differences. Future studies with larger post-treatment sample sizes will be important to specifically assess the performance of pooled urine testing strategies in low-intensity, post-treatment settings and to further refine optimal pooling volumes under these conditions.”

6.= L201 : “In each group, one positive sample was included, ………at random from the set of known positive specimens and was not chosen based on infection intensity.” This could lead to a bias. I am sure that an infected individual with 1000 eggs/10 mL in each pool and another with 10 eggs/mL will provide different diagnostic performance with the pool. The data in the current study would have been gaining added value if the pool size and volumes were analysis in the subset of individual with low and high infection based on UFT which is the gold standard.

**Response**: We thank the reviewer for this insightful comment regarding potential bias related to infection intensity within pooled samples. In response, we have reanalyzed the data by stratifying positive samples into light (<50 eggs/10 mL) and heavy (≥50 eggs/10 mL) infection intensity categories based on individual urine filtration microscopy results.

The subgroup analysis showed that the effects of both urine volume and pool size on the sensitivity of urine filtration microscopy (UFM) for detecting *S. haematobium* in pooled samples varied by infection intensity. The effect of urine volume was greater in heavy-intensity infections (≥50 eggs/10 mL; β = 0.06, SE = 0.018, p < 0.001) than in light-intensity infections (<50 eggs/10 mL; β = 0.03, SE = 0.006, p < 0.001). Similarly, the negative effect of increasing pool size on sensitivity was more pronounced in heavy-intensity infections (β = −0.06, SE = 0.010, p < 0.001) compared with light-intensity infections (β = −0.03, SE = 0.006, p < 0.001).

These findings have been incorporated into the Results section and support the reviewer’s concern that infection intensity modifies the diagnostic performance of pooled urine testing.

7. = L210: “One month after treatment, children who tested positive for S. haematobium infection using UFM in the field and were treated with praziquantel (40 mg/kg body weight) were re-examined using the same methodology as described above”. Where are the results and discussion related to this methodology?

**Response**: We would like the reviewer to see our response above.

8.=L86 = “A potential cost-saving strategy involves pooling individual samples and applying a cascaded pooled testing (CPT) strategy” Did you assess the costs of the different approaches? Otherwise, that can be discussed as a limit of the current work.

**Response**: We have not collected cost data in this study and discussed this as a limitation. The text in discussion reads as “Moreover, there is a lack of time and other cost-benefit analyses to delineate when pooling urine samples during a large-scale epidemiological survey of *S. haematobium* infection is indeed cost-saving. This is particularly relevant for the FC, which would require more time, personnel effort, and water for testing compared to conventional UFM, potentially limiting its feasibility for field deployment in resource-limited settings. This will support program managers and healthcare decision-makers in creating the most economical survey for rapidly monitoring MDA initiatives targeted at *S. haematobium* infection control.”

9.= Lines 105 – 107: “studies demonstrated that the pooled testing strategy allowed ….. prevalence when used in low intensity infection areas and when larger pools are tested.” It is difficult to find the data clearly supporting this statement.

**Response**: Thank you for this comment. We agree that the original statement was overly strong and did not clearly distinguish between infection intensity estimation and prevalence assessment. We have revised the paragraph to more cautiously reflect our previous findings, clarifying that pooled testing provides a rapid estimate of infection intensity, while sensitivity for prevalence estimation may decline in low-intensity settings, particularly with increasing pool size. The revised wording avoids overgeneralization and better aligns with the evidence presented in the cited studies.

10.= Line 440: “This study tested a low prevalence scenario by introducing single S. haematobium-positive specimens into each pool.” By diluting single positive urine sample in five does not mean that the infection intensity will become low! That will depend on the initial infection intensity in the given sample (10000, 5000 etc…).

**Response**: We thank the reviewer for this comment and appreciate the opportunity to clarify this point. Our intention in constructing pools with a single positive sample in this sentence was to simulate a low-prevalence setting, where only one infected individual is present within a group, rather than to model low-intensity infections. We agree that dilution of a positive urine sample within a pool does not necessarily alter the underlying infection intensity of that individual, which remains dependent on the initial egg count.

However, pooling inherently reduces the concentration of eggs per unit volume in the combined sample, which can affect diagnostic detectability, particularly for low-intensity infections. To address the reviewer’s concern, we have now conducted an additional subgroup analysis stratified by infection intensity (light vs. heavy), which confirms that diagnostic performance varies according to the initial intensity of infection within the pool.

The added results reads “A subgroup analysis showed that the effects of both urine volume and pool size on the sensitivity of urine filtration microscopy (UFM) for detecting *S. haematobium* in pooled samples varied by infection intensity. The effect of urine volume was greater in heavy-intensity infections (≥50 eggs/10 mL; β = 0.06, SE = 0.018, p < 0.001) than in light-intensity infections (<50 eggs/10 mL; β = 0.03, SE = 0.006, p < 0.001). Similarly, the negative effect of increasing pool size on sensitivity was more pronounced in heavy-intensity infections (β = −0.06, SE = 0.010, p < 0.001) compared with light-intensity infections (β = −0.03, SE = 0.006, p < 0.001).

**Reviewer #2**: The authors have satisfactorily addressed all comments and concerns raised in my initial review.

**Response**: We thank the reviewer for their positive assessment and for the constructive feedback provided during the initial review. We are pleased that the revisions have adequately addressed the comments and concerns raised.

**Reviewer #3**: The authors have fully addressed my comments and I find the manuscript to be greatly improved. Many thanks for your careful consideration of my suggestions, and congratulations on an important piece of work.

**Response**: We thank the reviewer for their positive assessment and for the constructive feedback provided during the initial review. We are pleased that the revisions have adequately addressed the comments and concerns raised.
