# Supplementary material for: Factors affecting detection and quantification of Schistosoma haematobium eggs in pooled urine samples
Source: PLoS Negl Trop Dis. 2026 Jun 1;20(6):e0014407. doi: 10.1371/journal.pntd.0014407 (PMC13245858; doi:10.1371/journal.pntd.0014407)
Supplement: Peer review history

# Peer review: Decision Letter 0

**DOI:** 10.1371/journal.pntd.0014407.r001

**Authors:** Richard Stewart Bradbury, Lucienne Tritten

11 Jan 2026

PNTD-D-25-01801

Detection and quantification of Schistosoma haematobium eggs in pooled urine samples

PLOS Neglected Tropical Diseases

Dear Dr. Degarege,

Thank you for submitting your manuscript to PLOS Neglected Tropical Diseases. After careful consideration, we feel that it has merit but does not fully meet PLOS Neglected Tropical Diseases's publication criteria as it currently stands. Therefore, we invite you to submit a revised version of the manuscript that addresses the points raised during the review process.

Please submit your revised manuscript by January 30th 2026. If you will need more time than this to complete your revisions, please reply to this message or contact the journal office at plosntds@plos.org. When you're ready to submit your revision, log on to https://www.editorialmanager.com/pntd/ and select the 'Submissions Needing Revision' folder to locate your manuscript file.

Please include the following items when submitting your revised manuscript:

* A letter that responds to each point raised by the editor and reviewer(s). You should upload this letter as a separate file labeled 'Response to Reviewers'. This file does not need to include responses to any formatting updates and technical items listed in the 'Journal Requirements' section below.

* A marked-up copy of your manuscript that highlights changes made to the original version. You should upload this as a separate file labeled 'Revised Manuscript with Track Changes'.

* An unmarked version of your revised paper without tracked changes. You should upload this as a separate file labeled 'Manuscript'.

If you would like to make changes to your financial disclosure, competing interests statement, or data availability statement, please make these updates within the submission form at the time of resubmission. Guidelines for resubmitting your figure files are available below the reviewer comments at the end of this letter.

We look forward to receiving your revised manuscript.

Kind regards,

Richard Stewart Bradbury, PhD

Section Editor

PLOS Neglected Tropical Diseases

Lucienne Tritten

Section Editor

PLOS Neglected Tropical Diseases

Shaden Kamhawi

co-Editor-in-Chief

PLOS Neglected Tropical Diseases

orcid.org/0000-0003-4304-636XX

Paul Brindley

co-Editor-in-Chief

PLOS Neglected Tropical Diseases

orcid.org/0000-0003-1765-0002

**Additional Editor Comments:**

The reviewers have raised several important points. The most pertinent of these is the need to consider anonymity of patient samples in low population villages, and some suggestions on how this may be done have been made. I encourage the authors to address all review comments and resubmit for further consideration.

**Journal Requirements:**

If the reviewer comments include a recommendation to cite specific previously published works, please review and evaluate these publications to determine whether they are relevant and should be cited. There is no requirement to cite these works unless the editor has indicated otherwise.

1) Please ensure that the CRediT author contributions listed for every co-author are completed accurately and in full.

At this stage, the following Authors/Authors require contributions: Abraham Degarege, Bruno Levecke, Christopher R Bilder, David M Brett-Major, Abebe Animut, Yohannes D Negash, M. Jana Broadhurst, Tzeyu D Michaud, and Berhanu Erko. Please ensure that the full contributions of each author are acknowledged in the "Add/Edit/Remove Authors" section of our submission form.

The list of CRediT author contributions may be found here: https://journals.plos.org/plosntds/s/authorship#loc-author-contributions

2) Please upload all main figures as separate Figure files in .tif or .eps format. For more information about how to convert and format your figure files please see our guidelines:

https://journals.plos.org/plosntds/s/figures

3) Some material included in your submission may be copyrighted. According to PLOSu2019s copyright policy, authors who use figures or other material (e.g., graphics, clipart, maps) from another author or copyright holder must demonstrate or obtain permission to publish this material under the Creative Commons Attribution 4.0 International (CC BY 4.0) License used by PLOS journals. Please closely review the details of PLOSu2019s copyright requirements here: PLOS Licenses and Copyright. If you need to request permissions from a copyright holder, you may use PLOS's Copyright Content Permission form.

Please respond directly to this email and provide any known details concerning your material's license terms and permissions required for reuse, even if you have not yet obtained copyright permissions or are unsure of your material's copyright compatibility. Once you have responded and addressed all other outstanding technical requirements, you may resubmit your manuscript within Editorial Manager.

Potential Copyright Issues:

i) Figure 1. Please confirm whether you drew the images / clip-art within the figure panels by hand. If you did not draw the images, please provide (a) a link to the source of the images or icons and their license / terms of use; or (b) written permission from the copyright holder to publish the images or icons under our CC BY 4.0 license. Alternatively, you may replace the images with open source alternatives. See these open source resources you may use to replace images / clip-art:

- https://commons.wikimedia.org

- https://openclipart.org/.

ii) Figure S1. Please (a) provide a direct link to the base layer of the map (i.e., the country or region border shape) and ensure this is also included in the figure legend; and (b) provide a link to the terms of use / license information for the base layer image or shapefile. We cannot publish proprietary or copyrighted maps (e.g. Google Maps, Mapquest) and the terms of use for your map base layer must be compatible with our CC BY 4.0 license.

Note: if you created the map in a software program like R or ArcGIS, please locate and indicate the source of the basemap shapefile onto which data has been plotted.

If your map was obtained from a copyrighted source please amend the figure so that the base map used is from an openly available source. Alternatively, please provide explicit written permission from the copyright holder granting you the right to publish the material under our CC BY 4.0 license.

If you are unsure whether you can use a map or not, please do reach out and we will be able to help you. The following websites are good examples of where you can source open access or public domain maps:

* U.S. Geological Survey (USGS) - All maps are in the public domain. (http://www.usgs.gov)

* PlaniGlobe - All maps are published under a Creative Commons license so please cite u201cPlaniGlobe, http://www.planiglobe.com, CC BY 2.0u201d in the image credit after the caption. (http://www.planiglobe.com/?lang=enl)

* Natural Earth - All maps are public domain. (http://www.naturalearthdata.com/about/terms-of-use/).

4) Kindly revise your competing statement in the online submission form to align with the journal's style guidelines: 'The authors declare that there are no competing interests.'

**Reviewers' comments:**

Reviewer's Responses to Questions

**Key Review Criteria Required for Acceptance?**

As you describe the new analyses required for acceptance, please consider the following:

**Methods**

-Are the objectives of the study clearly articulated with a clear testable hypothesis stated?

-Is the study design appropriate to address the stated objectives?

-Is the population clearly described and appropriate for the hypothesis being tested?

-Is the sample size sufficient to ensure adequate power to address the hypothesis being tested?

-Were correct statistical analysis used to support conclusions?

-Are there concerns about ethical or regulatory requirements being met?

Reviewer #1: See "summary and General comment"

Reviewer #2: I read the manuscript with great interest. The hypothesis is clearly stated, and the methods and procedures are well described. While I am not a statistical expert, the sample size and data analysis appear more than appropriate. The authors have an excellent track record in this field, which adds credibility to their work.

Reviewer #3: (No Response)

**********

**Results**

-Does the analysis presented match the analysis plan?

-Are the results clearly and completely presented?

-Are the figures (Tables, Images) of sufficient quality for clarity?

Reviewer #1: See "summary and General comment"

Reviewer #2: Yes; also the supplementary data file is highly appreciated.

Reviewer #3: (No Response)

**********

**Conclusions**

-Are the conclusions supported by the data presented?

-Are the limitations of analysis clearly described?

-Do the authors discuss how these data can be helpful to advance our understanding of the topic under study?

-Is public health relevance addressed?

Reviewer #1: See "summary and General comment"

Reviewer #2: Yes

Reviewer #3: (No Response)

**********

**Editorial and Data Presentation Modifications?**

Use this section for editorial suggestions as well as relatively minor modifications of existing data that would enhance clarity. If the only modifications needed are minor and/or editorial, you may wish to recommend “Minor Revision” or “Accept”.

Reviewer #1: (No Response)

Reviewer #2: I have only very minor comments.

Line 162: children who tested positive when tested at the collection site were treated with praziquantel. Please explain what happened with the children who were in first instance negative, but tested positive when the 10mL urine examination was repeated (Line 166) just before the experimental pooling.

Line 166: please indicate the time period (median and range) between urine collection and the experimental pooling.

Line 398 mentions the lack of a time-benefit and cost-benefit study. I would appreciate some additional words about the practicality of using the Fluke Catcher compared to Urine Filtration Microscopy.

Reviewer #3: (No Response)

**********

**Summary and General Comments**

Use this section to provide overall comments, discuss strengths/weaknesses of the study, novelty, significance, general execution and scholarship. You may also include additional comments for the author, including concerns about dual publication, research ethics, or publication ethics. If requesting major revision, please articulate the new experiments that are needed.

Reviewer #1: Author : Degarege et al.

Title : Detection and quantification of Schistosoma haematobium eggs in pooled urine

Samples

Journal : PLoS Negl Trop Dis

Reference : PNTD-D-25-01801

General Comment

The authors are dealing with an important topic which should have great impact in the era of schistosomiasis elimination and in the context of limited financial resources. Please find below comments and suggestions for improvement that the authors could trust in.

METHODS

= This study would have yielded more interesting results if the authors had not created conditions of low infection intensity by diluting the samples, but had instead considered the low, moderate, and heavy infections from their field sampling. This is especially relevant given the large sample size. Can the authors explain their choice to recreate conditions of low infection intensity?

= L17 – L22: When did you use the FC?

= Please combine both “Study rea” and “Study population” and named “Study area and population”

= L149: Please remove this “(=995+995+498 +100).”

=L144-L145: Not clear how the following numbers have been reaching “we needed to screen a minimum of 995 children in Afar, 995 children in Benishangul-Gumuz, and 498 in Gambella” Please, could describe more

= Which formular has been used to assess the sample size?

= L153-L155 “Then, the …. whose parents approved their participation”. When and how the consent from the parents have been collected.

=L155 – L157: “Between 10:00 am and 3:00 pm, children who came to the nearby health posts, schools, or open fields, were asked to bring a urine sample of at least in labeled 200 mL plastic containers” Please, which kind of sampling method was used? This part raises some concerns about how consent has been obtained from children’s parents.

= L161 “UFM results were shared with the children and their parents at the collection site” How this has been done since available cases nearby health posts, schools or open fields were targeted?

= L166 “We first re-tested 10 mL of the formalin-fixed urine samples from each child using UFM.” When has this been done after the fieldwork?

= Why has formalin been used?

= L173 “In each group, one positive sample was included, while the remaining samples were negative.” How has this sample been selected? What was the infection intensity of the selected one? What is the rationale behind this design of putting only one negative among the positives? It seems that this is far from the real world. Why not pool regardless of the infection status and record the type of samples pooled and consider the sample characteristics during the analysis?

=L234 – L236 “As the study population was members… institutional review board approved a waiver of signed consent for this study.” Then you clearly mentioned that above to help understanding.

= L254: “Figure 2. The prevalence and mean UEC” Which mean has been used? Arithmetic one or the geometric one.

Discussion

= L262 “Overall, the diagnostic sensitivity tends to increase as a function of the intensity level of infection and the volume of urine examined” Not sure if any investigation is required to support this statement. This is so obvious!!!

L350: “Overall, the diagnostic sensitivity tends to increase as a function of the intensity level of infection and the volume of urine examined” This assumption has never been highlighted in the ease the understanding of the overall work.

Reviewer #2: This manuscript provides valuable insights into whether pooling urine samples can facilitate population-based monitoring of Schistosoma haematobium infections. The text is well written, and the procedures are clearly described. The overall conclusion is highly relevant to the field.

Reviewer #3: This manuscript presents comparative results of two laboratory methods applied to over 2000 urine samples from school-aged children, as well as a series of pools made up of test-positive and test-negative samples. The subject is worthy of investigation and there is a large body of data available (which it is fantastic to see being supplied as supplementary information - thanks!), but the presentation of the manuscript and obvious gaps in the analysis as well as theoretical statistical background currently limit the applicability and impact of the work. I therefore have a number of comments to address before publication.

1. The description of the pooling process does not state if the urine samples (both the individual samples and resulting pools) were mixed before aliquots were taken, but since this would be standard practice when creating pooled samples in other applications I assume this is the case. Perfect mixing would imply that the number of eggs in each aliquot would be Poisson distributed (with mean given by the product of the number of eggs in the sample and proportion of the sample taken as the aliquot), amd therefore the number of eggs in the resulting pooled sample would also be Poisson distributed (as the sum of independent Poisson processes is itself Poisson). This gives a theoretical basis for the expected distribution of eggs (based on the observed egg counts from the individual samples) that would be very useful to compare to the actual number of observed eggs, but this analysis is currently completely absent. This would greatly add to the paper: if the observed distribution closely matches the theoretical expectation, then this would support the use of computer simulation to analyse different pooling strategies. On the other hand, any departures from the theoretical expectation can be taken as evidence that the mixing process is imperfect and/or the egg recovery of the laboratory methods is biased.

2. There is a substantial body of literature on the utility (and approaches to) pooling for diagnosis, going all the way back to the 1940's. A lot of the lessons learned from this literature would be useful here, I think - or at the very least it should be acknowledged. You could usefully start with https://doi.org/10.1016/j.cmi.2021.04.007 and go from there.

3. Given that you have paired samples from each laboratory method applied to each (pooled) sample, I was disappointed not to see a direct comparison. For example, a paired qq plot (perhaps separated by volume of urine) would show if the observed increased probability of observing zero eggs for one test was mirrored for other egg values - i.e. is the bias between the tests consistent, or dependent on the number of eggs in the sample?

4. The logistic regression is one possible analysis, but did the authors consider fitting an over-dispersed count distribution to the data, with pool size as a fixed effect? I would expect the over-dispersion to depend on the volume of urine used, so this should probably be a stratified analysis and the over-dispersion parameters compared. This is related to point (1) above (and could be used as an analysis method against which to compare the expected distribution). It would also be useful to fit a similar model (perhaps using a zero-inflated over-dispersed count model) to the individual data, to concurrently estimate risk factors for intensity and true prevalence of infection.

I also found the following more minor issues:

- I note that the individual samples were classified as positive/negative (for pooling) based on UFM test only - could this have in some way affected the comparison of UFM and FC? I don't think so, but it would be good to see this considered explicitly.

- I think that Figure 2A and 2B would be more informative as histograms or similar - then we can see the full distribution (or perhaps adding a histogram for each region, or similar, if you don't want to have too many histograms).

- Figures 3 and 4 are far too large considering how little information they convey - and is figure 4 not just model predictions? These can be replaced with something more useful, I think.

- Please correctly cite both R and the R packages (e.g. ggplot2) that you use for your analysis

- Although it is highly commendable to include the raw data as supplementary material, I am concerned about the information (age/sex/village) not meeting the standard required for anonymity (e.g. if there are fewer than 5 children of given age and gender in a specific village, we would typically consider this to be potentially re-identifiable personal [sensitive, as it is health-related] data). Would it not be prudent to anonymise the village names, and possibly also group the ages to ensure that all combinations of identifying features have at least 5 individuals?

**********

PLOS authors have the option to publish the peer review history of their article (what does this mean?). If published, this will include your full peer review and any attached files.

If you choose “no”, your identity will remain anonymous but your review may still be made public.

**Do you want your identity to be public for this peer review?** For information about this choice, including consent withdrawal, please see our Privacy Policy.

Reviewer #1: **Yes:** Jean T. Coulibaly

Reviewer #2: No

Reviewer #3: No

**Figure resubmission:**

While revising your submission, we strongly recommend that you use PLOS’s NAAS tool (https://ngplosjournals.pagemajik.ai/artanalysis) to test your figure files. NAAS can convert your figure files to the TIFF file type and meet basic requirements (such as print size, resolution), or provide you with a report on issues that do not meet our requirements and that NAAS cannot fix.

After uploading your figures to PLOS’s NAAS tool - https://ngplosjournals.pagemajik.ai/artanalysis, NAAS will process the files provided and display the results in the "Uploaded Files" section of the page as the processing is complete. If the uploaded figures meet our requirements (or NAAS is able to fix the files to meet our requirements), the figure will be marked as "fixed" above. If NAAS is unable to fix the files, a red "failed" label will appear above. When NAAS has confirmed that the figure files meet our requirements, please download the file via the download option, and include these NAAS processed figure files when submitting your revised manuscript.

**Reproducibility:**

To enhance the reproducibility of your results, we recommend that authors of applicable studies deposit laboratory protocols in protocols.io, where a protocol can be assigned its own identifier (DOI) such that it can be cited independently in the future. Additionally, PLOS ONE offers an option to publish peer-reviewed clinical study protocols. Read more information on sharing protocols at https://plos.org/protocols?utm_medium=editorial-email&utm_source=authorletters&utm_campaign=protocols

# Author comment: Author response to Decision Letter 1

**DOI:** 10.1371/journal.pntd.0014407.r002

10 Mar 2026

Attachment Submitted filename: Response to Reviewers Comments.docx

# Peer review: Decision Letter 1

**DOI:** 10.1371/journal.pntd.0014407.r003

**Authors:** Luc E. Coffeng, Lucienne Tritten

28 Apr 2026

PNTD-D-25-01801R1

Detection and quantification of Schistosoma haematobium eggs in pooled urine samples

PLOS Neglected Tropical Diseases

Dear Dr. Degarege,

Thank you for submitting your manuscript to PLOS Neglected Tropical Diseases. After careful consideration, we feel that it has merit but does not fully meet PLOS Neglected Tropical Diseases's publication criteria as it currently stands. Therefore, we invite you to submit a revised version of the manuscript that addresses the points raised during the review process.

Please submit your revised manuscript by May 27, 2026. If you will need more time than this to complete your revisions, please reply to this message or contact the journal office at plosntds@plos.org. When you're ready to submit your revision, log on to https://www.editorialmanager.com/pntd/ and select the 'Submissions Needing Revision' folder to locate your manuscript file.

-->Please include the following items when submitting your revised manuscript:

* A letter that responds to each point raised by the editor and reviewer(s). You should upload this letter as a separate file labeled 'Response to Reviewers'. This file does not need to include responses to any formatting updates and technical items listed in the 'Journal Requirements' section below.

-->* A marked-up copy of your manuscript that highlights changes made to the original version. You should upload this as a separate file labeled 'Revised Manuscript with Track Changes'.

* An unmarked version of your revised paper without tracked changes. You should upload this as a separate file labeled 'Manuscript'.

-->If you would like to make changes to your financial disclosure, competing interests statement, or data availability statement, please make these updates within the submission form at the time of resubmission. Guidelines for resubmitting your figure files are available below the reviewer comments at the end of this letter.

We look forward to receiving your revised manuscript.

Kind regards,

Luc E. Coffeng, MD PhD

Academic Editor

-->PLOS Neglected Tropical Diseases

Lucienne Tritten

Section Editor

PLOS Neglected Tropical Diseases

Shaden Kamhawi

co-Editor-in-Chief

PLOS Neglected Tropical Diseases

orcid.org/0000-0003-4304-636XX

Paul Brindley

co-Editor-in-Chief

PLOS Neglected Tropical Diseases

orcid.org/0000-0003-1765-0002

**Editor Comments :**

Please perform a last check of the manuscript for spelling. Examples of typos are "aliqute" and "positisve".

In addition, please consider rephrasing some of the following statements, which seem to cause confusion with some of the reviewers:

LINE 123: “The aim of our study was to explore when ……. S. haematobium infections in the context of an MDA program.”

--> Reviewer #1:: What do you mean by “When” I did not find results related to that “when” in the MS

LINE 132: “Urine samples were collected at baseline and one month after praziquantel was administered to children infected with the parasite based on urine testing results.”

--> Reviewer #1: Did you not find the results related to posttreatment performance of the pooling and which volume if the best one?

LINE 438: Similarly, since S. haematobium eggs are unevenly distributed in urine samples [3,4],

using a larger volume may help minimize these variations (i.e., dilute random fluctuations in egg

concentration), making it easier to detect low-intensity infections in pooled samples.

--> Editor: Avoid "dilute", which seems to refer to literal dilution, whereas I presume the authors actually mean "reduce" or more specifically "reduce the probability of finding zero eggs"? If this is not the intended message, I'm not sure I understand.

**********

Note:If the reviewer comments include a recommendation to cite specific previously published works, please review and evaluate these publications to determine whether they are relevant and should be cited. There is no requirement to cite these works unless the editor has indicated otherwise.

**Reviewers' Comments:**

**Summary and General Comments**

Use this section to provide overall comments, discuss strengths/weaknesses of the study, novelty, significance, general execution and scholarship. You may also include additional comments for the author, including concerns about dual publication, research ethics, or publication ethics. If requesting major revision, please articulate the new experiments that are needed.

Reviewer #1:  Major comment

= The title: “Detection and quantification of Schistosoma haematobium eggs in pooled urine

samples” Must be revised with consideration to the content of the MS

In the abstract:

= “(=0.01, p=0.021) or pool size (=-0.02, p=0.007)”. What is front of the “=” otherwise that does not make sense

= Lack of clear take away message in the conclusion and did not include all the methodologies and key results regarding the initial hypothesis of the current work. Which pool or volume is the good one and why? The way the authors concluded here seems to be more focused on already known findings, nothing new has been provided based on their own work

= L123 “The aim of our study was to explore when ……. S. haematobium infections in the context of an MDA program.” What do you mean by “When” I did not find results related to that “when” in the MS

= L132 “Urine samples were collected at baseline and one month after praziquantel was administered to children infected with the parasite based on urine testing results.” Did you not find the results related to posttreatment performance of the pooling and which volume if the best one?

= L201 : “In each group, one positive sample was included, ………at random from the set of known positive specimens and was not chosen based on infection intensity.” This could lead to a bias. I am sure that an infected individual with 1000 eggs/10 mL in each pool and another with 10 eggs/mL will provide different diagnostic performance with the pool. The data in the current study would have been gaining added value if the pool size and volumes were analysis in the subset of individual with low and high infection based on UFT which is the gold standard.

= L210: “One month after treatment, children who tested positive for S. haematobium infection using UFM in the field and were treated with praziquantel (40 mg/kg body weight) were re-examined using the same methodology as described above”. Where are the results and discussion related to this methodology?

=L86 = “A potential cost-saving strategy involves pooling individual samples and applying a cascaded87

pooled testing (CPT) strategy” Did you assess the costs of the different approaches? Otherwise, that can be discussed as a limit of the current work

= Lines 105 – 107: “studies demonstrated that the pooled testing strategy allowed ….. prevalence when used in low intensity infection areas and when larger pools are tested.” It is difficult to find the data clearly supporting this statement.

= Line 440: “This study tested a low prevalence scenario by introducing single S. haematobium-positive specimens into each pool.” By diluting single positive urine sample in five does not mean that the infection intensity will become low! That will depend on the initial infection intensity in the given sample (10000, 5000 etc…)

Reviewer #2:  The authors have satisfactorily addressed all comments and concerns raised in my initial review.

Reviewer #3:  The authors have fully addressed my comments and I find the manuscript to be greatly improved. Many thanks for your careful consideration of my suggestions, and congratulations on an important piece of work.

**********

**Do you want your identity to be public for this peer review?** For information about this choice, including consent withdrawal, please see our Privacy Policy.

Reviewer #1: **Yes:** Jean T. Coulibaly

Reviewer #2: No

Reviewer #3: No

[NOTE: If reviewer comments were submitted as an attachment file, they will be attached to this email and accessible via the submission site. Please log into your account, locate the manuscript record, and check for the action link "View Attachments". If this link does not appear, there are no attachment files.]

**Figure resubmission:**

While revising your submission, we strongly recommend that you use PLOS’s NAAS tool (https://ngplosjournals.pagemajik.ai/artanalysis) to test your figure files. NAAS can convert your figure files to the TIFF file type and meet basic requirements (such as print size, resolution), or provide you with a report on issues that do not meet our requirements and that NAAS cannot fix.

After uploading your figures to PLOS’s NAAS tool - https://ngplosjournals.pagemajik.ai/artanalysis, NAAS will process the files provided and display the results in the "Uploaded Files" section of the page as the processing is complete. If the uploaded figures meet our requirements (or NAAS is able to fix the files to meet our requirements), the figure will be marked as "fixed" above. If NAAS is unable to fix the files, a red "failed" label will appear above. When NAAS has confirmed that the figure files meet our requirements, please download the file via the download option, and include these NAAS processed figure files when submitting your revised manuscript.

**Reproducibility:**

-->To enhance the reproducibility of your results, we recommend that authors of applicable studies deposit laboratory protocols in protocols.io, where a protocol can be assigned its own identifier (DOI) such that it can be cited independently in the future. Additionally, PLOS ONE offers an option to publish peer-reviewed clinical study protocols. Read more information on sharing protocols at https://plos.org/protocols?utm_medium=editorial-email&utm_source=authorletters&utm_campaign=protocols

# Author comment: Author response to Decision Letter 2

**DOI:** 10.1371/journal.pntd.0014407.r004

5 May 2026

Attachment Submitted filename: Response to editors and reviewers comments .docx

# Editor report: Decision Letter 2

**DOI:** 10.1371/journal.pntd.0014407.r005

**Authors:** Luc E. Coffeng, Lucienne Tritten

25 May 2026

Dear Degarege,

We are pleased to inform you that your manuscript 'Factors affecting detection and quantification of *Schistosoma haematobium* eggs in pooled urine samples' has been provisionally accepted for publication in PLOS Neglected Tropical Diseases.

Before your manuscript can be formally accepted you will need to complete some formatting changes, which you will receive in a follow up email. A member of our team will be in touch with a set of requests.

Please note that your manuscript will not be scheduled for publication until you have made the required changes, so a swift response is appreciated.

IMPORTANT: The editorial review process is now complete. PLOS will only permit corrections to spelling, formatting or significant scientific errors from this point onwards. Requests for major changes, or any which affect the scientific understanding of your work, will cause delays to the publication date of your manuscript.

Should you, your institution's press office or the journal office choose to press release your paper, you will automatically be opted out of early publication. We ask that you notify us now if you or your institution is planning to press release the article. All press must be co-ordinated with PLOS.

Thank you again for supporting Open Access publishing; we are looking forward to publishing your work in PLOS Neglected Tropical Diseases.

Best regards,

Luc E. Coffeng, MD PhD

Academic Editor

PLOS Neglected Tropical Diseases

Lucienne Tritten

Section Editor

PLOS Neglected Tropical Diseases

Shaden Kamhawi

co-Editor-in-Chief

PLOS Neglected Tropical Diseases

orcid.org/0000-0003-4304-636XX

Paul Brindley

co-Editor-in-Chief

PLOS Neglected Tropical Diseases

orcid.org/0000-0003-1765-0002

***********************************************************

p.p1 {margin: 0.0px 0.0px 0.0px 0.0px; line-height: 16.0px; font: 14.0px Arial; color: #323333; -webkit-text-stroke: #323333}span.s1 {font-kerning: none

# Editor report: Acceptance letter

**DOI:** 10.1371/journal.pntd.0014407.r006

**Authors:** Luc E. Coffeng, Lucienne Tritten

Dear Dr Degarege,

We are delighted to inform you that your manuscript, "Factors affecting detection and quantification of Schistosoma haematobium eggs in pooled urine samples," has been formally accepted for publication in PLOS Neglected Tropical Diseases.

We have now passed your article onto the PLOS Production Department who will complete the rest of the publication process. All authors will receive a confirmation email upon publication.

The corresponding author will soon be receiving a typeset proof for review, to ensure errors have not been introduced during production. Please review the PDF proof of your manuscript carefully, as this is the last chance to correct any scientific or type-setting errors. Please note that major changes, or those which affect the scientific understanding of the work, will likely cause delays to the publication date of your manuscript. Note: Proofs for Front Matter articles (Editorial, Viewpoint, Symposium, Review, etc...) are generated on a different schedule and may not be made available as quickly.

Soon after your final files are uploaded, the early version of your manuscript will be published online unless you opted out of this process. The date of the early version will be your article's publication date. The final article will be published to the same URL, and all versions of the paper will be accessible to readers.

For Research Articles, you will receive an invoice from PLOS for your publication fee after your manuscript has reached the completed accept phase. If you receive an email requesting payment before acceptance or for any other service, this may be a phishing scheme. Learn how to identify phishing emails and protect your accounts at https://explore.plos.org/phishing.

Thank you again for supporting open-access publishing; we are looking forward to publishing your work in PLOS Neglected Tropical Diseases.

Best regards,

Shaden Kamhawi

co-Editor-in-Chief

PLOS Neglected Tropical Diseases

Paul Brindley

co-Editor-in-Chief

PLOS Neglected Tropical Diseases
